# A novel nasal powder formulation of glucagon: toxicology studies in animal models

**DOI:** 10.1186/s40360-015-0026-9

**Published:** 2015-10-26

**Authors:** Frederick E. Reno, Patrick Normand, Kevin McInally, Sherwin Silo, Patricia Stotland, Myriam Triest, Dolores Carballo, Claude Piché

**Affiliations:** 130 Macaw Lane, Merritt Island, FL 32952 USA; ITR Laboratories Canada Inc. (ITR), 19601 Clark Graham Blvd, Baie d’Urfe, Quebec Canada; CiToxLAB North America, 445 Armand-Frappier Blvd, Laval, Québec Canada; Locemia Solutions ULC., 8505 Dalton, Montreal, QC Canada

**Keywords:** Diabetes, Glucagon, Hypoglycemia, Insulin, Intranasal, Peptide hormones

## Abstract

**Background:**

Glucagon nasal powder (GNP), a novel intranasal formulation of glucagon being developed to treat insulin-induced severe hypoglycemia, contains synthetic glucagon (10 % *w/w*), beta-cyclodextrin, and dodecylphosphocholine. The safety of this formulation was evaluated in four studies in animal models.

**Methods:**

The first study evaluated 28-day sub-chronic toxicology in rats treated intranasally with 1 and 2 mg of GNP/day (0.1 and 0.2 mg glucagon/rat/day). The second study evaluated 28-day sub-chronic toxicology in dogs administered 20 and 40 mg of formulation/dog/day (2 and 4 mg glucagon/dog/day) intranasally. A pulmonary insufflation study assessed acute toxicology following intra-tracheal administration of 0.5 mg of GNP (0.05 mg glucagon) to rats. Local tolerance to 30 mg of GNP (equivalent to 3 mg glucagon, the final dose for humans) was tested through direct administration into the eyes of rabbits.

**Results:**

There were no test article-related adverse effects on body weight and/or food consumption, ophthalmology, electrocardiography, hematology, coagulation parameters, clinical chemistry, urinalysis, or organ weights, and no macroscopic findings at necropsy in any study. In rats, direct intra-tracheal insufflation at a dose of 0.5 mg of GNP/rat (0.05 mg glucagon/rat) did not result in adverse clinical, macroscopic, or microscopic effects. In dogs, the only adverse findings following sub-chronic use were transient (<30 s) salivation and sneezing immediately post-treatment and mild to moderate reversible histological changes to the nasal mucosa. Daily dosing over 28 days in rats resulted in mild to moderate, unilateral or bilateral erosion/ulceration of the olfactory epithelium, frequently with minimal to mild, acute to sub-acute inflammation of the lamina propria at the dorsal turbinates of the nasal cavity in 2/10 males and 3/10 females in the high-dose group (0.2 mg glucagon/day). These lesions resolved completely over 14 days. Histological examination of tissues from both sub-chronic studies in dogs and rats revealed no microscopic findings. In rabbits, clinical observations noted in the GNP-treated eye and/or surrounding areas included ≥1 of the following: clear discharge, red conjunctiva, partial closure, and swelling of the peri-orbital area, which correlated with erythema and edema noted during ocular observations and grading.

**Discussion:**

The studies reported here revealed no safety concerns associated with GNP in animal models. Studies published earlier have highlighted the local safety profile of intranasally administered cyclodextrins (a component of GNP). The choline group, the phosphate group, and the saturated 12-carbon aliphatic chain that are present in the dodecylphosphocholine excipient used in GNP are all present in the phospholipids and lecithins seen ubiquitously in mammalian cell membranes and are unlikely to pose safety concerns; this notion is supported by several studies conducted by the authors that revealed no safety concerns. Taken together, these results suggest that intranasal delivery of GNP holds promise as a future rescue medication for use by caregivers to treat insulin-induced hypoglycemic episodes in patients with type 1 or type 2 diabetes.

**Conclusion:**

This novel drug product is well tolerated in animal models.

**Electronic supplementary material:**

The online version of this article (doi:10.1186/s40360-015-0026-9) contains supplementary material, which is available to authorized users.

## Background

Diabetes mellitus has reached epidemic proportions in much of the western world and is a serious and growing public health concern in many developing economies. Globally, there are approximately 285 million people with diabetes and that number is expected to reach 438 million by 2030.

Diabetes complications are usually associated with chronically elevated blood glucose levels, which result in heart, kidney, and eye diseases, amputations, and neurological impairment. Unfortunately, there are very real and serious complications associated with use of medications used to treat diabetes-related hyperglycemia. One of the most common complications of treatment to reduce blood sugar levels is hypoglycemia, most frequently seen in patients being treated with insulin (i.e., all patients with type 1 diabetes as well as patients with type 2 diabetes on insulin), but also in patients with type 2 diabetes receiving insulin secretagogue (sulfonylurea and meglitinides) treatment. Indeed, if it were not for the barrier of hypoglycemia, people with diabetes could probably have normal blood glucose levels and thus avoid the complications associated with hyperglycemia [1].

Depending on the severity of the episode, hypoglycemia causes a wide range of physical problems ranging from weakness, dizziness, sweating, and hunger to more serious symptoms including blurred vision, behavior change, seizures, coma and even death. In addition to the physical effects of hypoglycemia, there are significant psychological effects including fear of another episode, high levels of anxiety, and low levels of overall happiness that adversely affect glucose control and quality of life [2].

Based on reviews of the subject, patients with type 1 diabetes experience an average of one episode of severe temporarily disabling hypoglycemia each year [3, 4]. Although persons with type 2 diabetes are generally less susceptible to severe hypoglycemia, the incidence of severe hypoglycemia is significant with annual incidence rates varying from 0.02 to 0.35 episodes per patient per year [5]. However, as the disease progresses, patients with type 2 diabetes frequently show similar susceptibility to severe hypoglycemia as seen in patients with type 1 diabetes [6, 7].

According to the Standards of Medical Care in Diabetes of the American Diabetes Association (ADA), severe hypoglycemia in a conscious person should be treated by the oral ingestion of 15–20 g carbohydrate, preferably as glucose tablets or equivalent [8]. For severe hypoglycemia in an unconscious individual in the home setting, the recommended treatment is 1 mg glucagon by injection. For severe hypoglycemia in an unconscious individual in the presence of professional medical assistance and intravenous access, 10–25 g intravenous dextrose is recommended. In all cases, once the hypoglycemia has been reversed, the patient should be given oral carbohydrates to prevent repeated hypoglycemia.

Glucagon is a highly effective treatment for severe hypoglycemia outside and within the hospital setting. Because glucagon is unstable in solution, commercially available emergency glucagon kits consist of lyophilized glucagon powder that must be mixed with a diluent immediately prior to injection. This procedure can be complex, intimidating, and prone to potentially serious errors, especially in an emergency situation for a caregiver, companion, or stranger who is not trained in reconstitution and injection techniques.

**Fig. 1:**
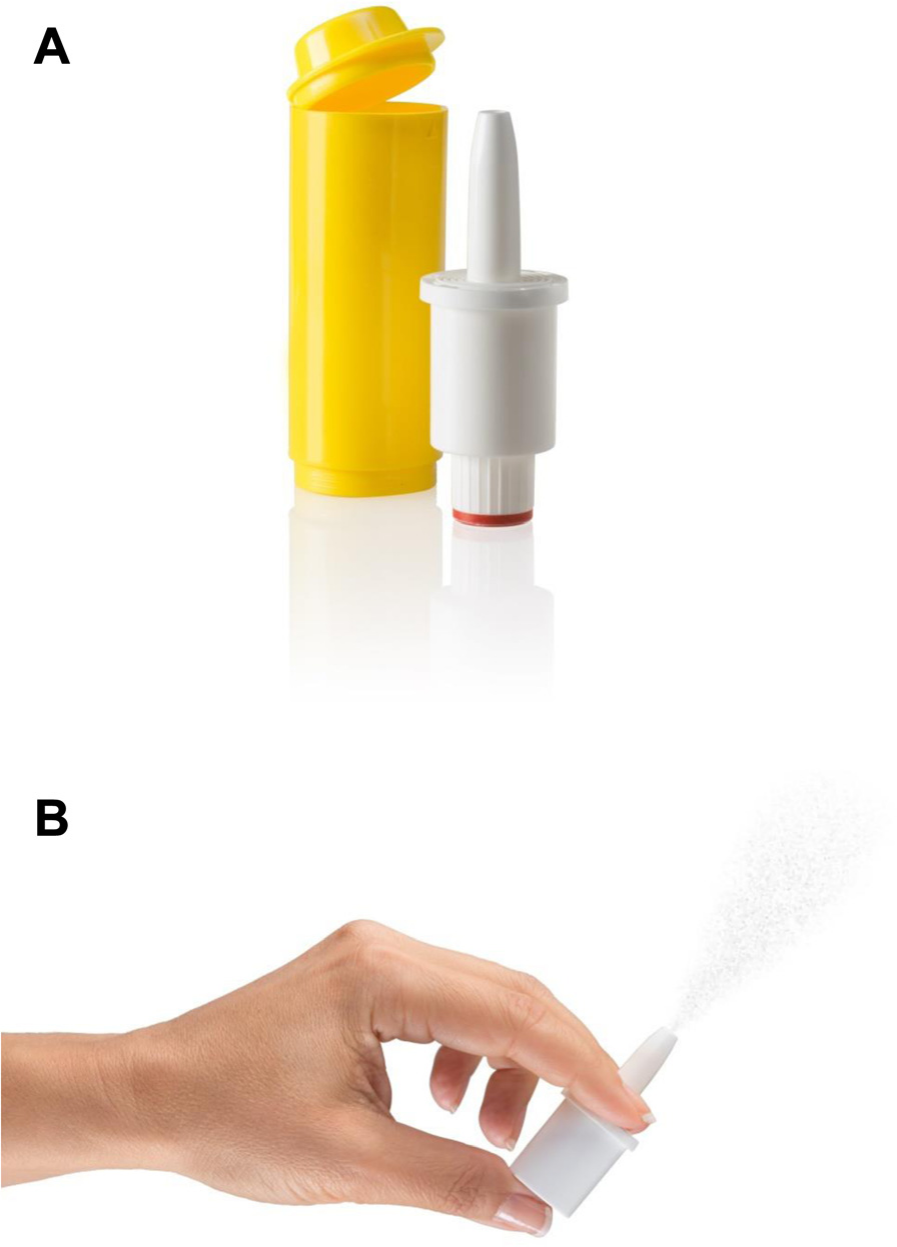
(**a**) GNP dosing device and carrying tube (**b**) GNP dosing device at activation

Glucagon nasal powder (GNP; previously referred to as AMG504-1) is a novel, nasally administered glucagon powder formulation being developed for the treatment of severe hypoglycemia, a commonly encountered life-threatening emergency in which the patient, typically an insulin-using diabetic, requires third-party assistance to correct the hypoglycemia. The drug is packaged within a very simple and user-friendly, single-use, nasal powder dosing device that delivers its entire contents into the patient’s nose when it is activated by pushing on the bottom of the dispenser (Fig. 1). The performance of the device is patient-independent in that there is no need to inhale (drug is absorbed from the nasal cavity) and the device performs as expected regardless of the orientation of the device (i.e., right side up or rotated 180°). This product may simplify treatment of severe hypoglycemia for non-medical caregivers of persons with diabetes.

GNP contains synthetic glucagon (10 % *w/w*) as well as beta-cyclodextrin and dodecylphosphocholine (DPC). Of the three ingredients in GNP, extensive safety and toxicology data and a long history of clinical use have been generated for glucagon and beta-cyclodextrin. The third ingredient, DPC, contains a choline group, a phosphate group, and a saturated aliphatic chain that is 12 carbons in length. As all three moieties are present in phospholipids and lecithins ubiquitous in mammalian cell membranes [9], significant adverse effects associated with DPC were not expected when it was chosen as the excipient.

The toxicology program for GNP consists of three components. The actual formulation (i.e., combination of glucagon, beta-cyclodextrin, and DPC) selected for clinical trials was studied to evaluate local and systemic effects of the drug product. A second series of studies, developed in accordance with the United States FDA’s Guidance for Industry: Nonclinical Studies for the Development of Pharmaceutical Excipients, was conducted to provide additional safety information and supplement the safety profile of DPC. A third set of studies was conducted to evaluate the *in vitro* cytotoxicity, skin sensitization, and intra-cutaneous reactivity of the materials used in the actuator delivery device. We now report the findings of four toxicology studies conducted in animal models to evaluate the safety of the actual formulation of this product. The findings of the toxicology studies with DPC and the materials used in the delivery device are being reported separately.

## Methods

The four toxicology studies reported here included the following: a 28-day sub-chronic toxicology study in rats evaluating the safety of GNP in solution at 0.1 and 0.2 mg/rat/day (approximately 0.4 and 0.8 mg/kg/day), a 28-day sub-chronic toxicology study in dogs that evaluated the safety of GNP in powder form at 2 and 4 mg glucagon/dog/day (i.e., 20 mg and 40 mg GNP/dog/day, approximately 0.2 and 0.4 mg/kg/day), an acute toxicology study in rats administered 0.5 mg GNP intra-tracheally (i.e., 0.05 mg glucagon), and a local tolerance study in rabbits in which 30 mg GNP (i.e., 3 mg glucagon) was administered directly in the eye (Table 1). The first three studies (the 28-day sub-chronic toxicology studies in rats and dogs, and the acute toxicology study in rats) were conducted at the facilities of ITR Laboratories Canada Inc, Baie d’Urfe, Québec, Canada, while the last study (local tolerance study in rabbits) was conducted at the facilities of CiToxLab North America, Laval (Québec), Canada. The test species were chosen because they are the species recommended for these types of studies by the regulatory authorities.

**Table 1:** Pivotal toxicology studies conducted with the GNP drug product

Study type	Species/N/Gender	Test articles and dosage
28-day sub-chronic toxicology	Rat/71M/71F	Saline, placebo liquid, GNP ingredients in solution at 0.1 and 0.2 mg/rat/day for 28 days
28-day sub-chronic toxicology	Dog/16M/16F	Saline, placebo powder, GNP at 2 and 4 mg/dog/day for 28 days
Acute toxicology	Rat/16M/16F	Air placebo control, GNP at 0.5 mg intra-tracheally
Acute toxicology	Rabbit	30-mg drug product administered directly in eye

*M* male, *F* female

The protocols for the studies conducted at the facilities of ITR Laboratories Canada Inc were reviewed and assessed by the Animal Care Committee (ACC) of ITR. All animals used in these studies were cared for in accordance with the principles outlined in the current “Guide to the Care and Use of Experimental Animals” as published by the Canadian Council on Animal Care and the “Guide for the Care and Use of Laboratory Animals”, a National Institutes of Health (NIH) publication. The studies did not unnecessarily duplicate previous experiments. Procedures involving the care and use of animals in the study conducted at the facilities of CiToxLab North America were reviewed and approved by the Institutional Animal Care and Use Committee (IACUC) prior to conduct. During the study, the care and use of animals was conducted in accordance with the principles outlined in the current Guidelines published by the Canadian Council on Animal Care and the Guide for the Care and Use of Laboratory Animals, a National Research Council (NRC) publication. The CiToxLAB North America facility is accredited by the Canadian Council on Animal Care and AAALAC.

Sprague–Dawley Crl:CD (SD) rats (*Rattus norvegicus*) and male New Zealand white rabbits (*Oryctolagus cuniculus*) were obtained from Charles River Canada Inc. (St-Constant, Quebec, Canada). Beagle dogs were obtained from Marshall BioResources, Inc. (North Rose, New York, USA). On arrival, all animals were weighed and subjected to a detailed physical examination by the Clinical Veterinarian to ensure satisfactory health status. Each animal was uniquely identified. Animals were fed and housed using standard protocols, and allowed an appropriate acclimation period between receipt of animals and start of treatment.

Detailed descriptions of methodology used in these studies are provided in the Supplement to this article Additional file 1.

## Results

### Twenty-eight-day intra-nasal toxicity followed by a 14-day recovery period in rats

#### Formulation analysis

The glucagon content in the test article formulation for Day 1 and for Day 28 was 5.7 and 5.8 mg/mL, respectively, therefore, within at least 2 % of the desired value of 5.7 mg glucagon/mL. The absence of glucagon was also confirmed in both control articles on both Days 1 and 28.

#### Mortality

Placebo-control animals 1006B and 1518D were found dead after dosing was performed on Days 13 and 15, respectively. A necropsy of animal 1006B indicated the cause of death was most likely physical trauma and not related to placebo control as no clinical signs were observed prior to the animal death. There were no gross findings for animal 1518D and, as a toxicokinetic (TK) animal, a microscopic examination was not performed. There were no deaths in saline or test-article groups.

#### Clinical signs

There were no adverse clinical signs related to treatment with GNP. All clinical signs noted were considered incidental and not related to the administration of GNP since they were sporadic, infrequent, and/or present in control animals at a similar frequency and/or incidence.

#### Body weight

There were no body weight changes related to treatment with GNP. All body weight changes were considered incidental and not related to the administration of GNP.

#### Food consumption

There were slight increases in food consumption for the weekly periods between Days 8 and 15 and between Days 15 and 22. However, these were observed in all groups and were not considered treatment-related.

#### Ophthalmoscopy

No treatment-related findings were noted during the course of this study and the observations recorded were incidental in origin and to be expected in this type of animal.

#### Toxicokinetics

**Fig. 2:**
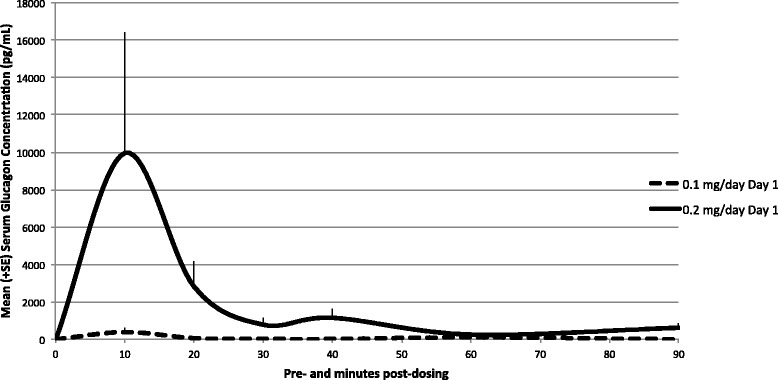
Mean (+ SE) glucagon serum concentration profiles in rats following a single intra-nasal administration—Day 1. Glucagon concentrations on Day 1 (linear scale, gender combined). Levels were below the lower limit of quantification (LLOQ, 200 pg/mL) in all samples collected from rats in the control groups and low- and high-dose groups prior to dosing on Day 1. Following the first intra-nasal drug administration on Day 1, only four of 12 animals in the low-dose GNP group displayed levels of glucagon above the LLOQ. Following a single high-dose GNP intra-nasal administration, all animals (12 of 12) displayed levels above LLOQ on at least one occasion, and mean glucagon concentration levels increased quickly in rat serum to reach peak levels within 10 min and declined over the sampling interval

Levels of glucagon were below the lower limit of quantification (LLOQ, 200 pg/mL) in all serum samples collected from rats in the control groups, and low- and high-dose groups prior to dosing on Day 1. Following the first intra-nasal (IN) administration on Day 1, only 4 of 12 animals in the low-dose group displayed levels of glucagon above LLOQ while 12 of 12 animals in the high-dose group displayed levels above LLOQ on at least one occasion. The mean glucagon concentration levels increased quickly in rat serum to reach peak levels within 10 min and declined over the sampling interval in both treated groups, but peak levels were higher in the high-dose group. Following Day 1 administration, mean systemic exposure (AUC_0-t_) to glucagon increased with dose (8095 vs. 172,893 pg.min/mL, for the low- and high-dose groups, respectively) and mean peak glucagon was observed 10 min after dosing and increased with dose (390 vs. 9961 pg/mL, for the low- and high-dose groups, respectively) (Fig. 2, Table 2).

**Table 2:** Summary toxicokinetic parameters of glucagon in rats (gender combined)

Day	Group	Dose level of glucagon (mg/day)	Mean (± SE)				
			AUC_0–t_ (pg min/mL)	AUC_0–90_ (pg min/mL)	C_max_ (pg/mL)	t_max_ (min)	R_A_
1	Low dose	0.1	8095 ± 3266	8095 ± 3266	390 ± 240	10.0	NC
	High dose	0.2	172,893 ± 72,774	172,893 ± 72,774	9961 ± 6443	10.0	NC
28	Low dose	0.1	43,036 ± 21,047	43,036 ± 21,047	988 ± 851	60.0	5.31
	High dose	0.2	60,073 ± 20,639	60,073 ± 20,639	2109 ± 1464	20.0	0.347

*NC* not calculated

**Fig. 3:**
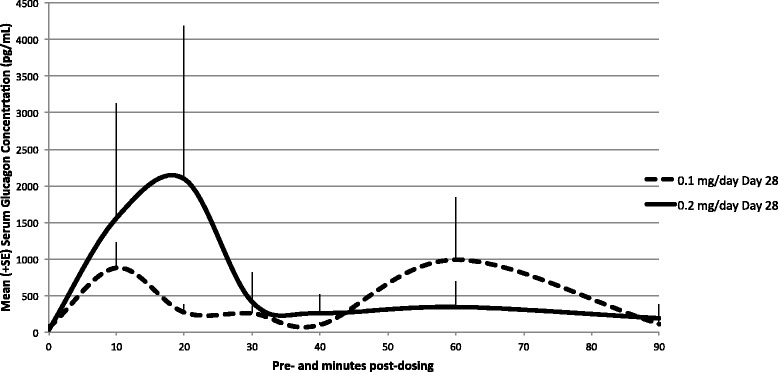
Mean (+ SE) glucagon serum concentration profiles in rats following 28 consecutive days of daily intra-nasal administration—Day 28. Glucagon concentrations on Day 28 (linear scale, gender combined). All samples collected from placebo-control liquid and saline-control animals displayed undetectable levels of glucagon with the exception of two samples at 20 min post-dose, one from the placebo-control liquid group (animal 1016D, 8644 pg/mL) and one from the saline-control group (animal 2011D, 45 pg/mL). Following 28 days of consecutive daily dosing, all pre-dose samples collected from low- and high-dose groups were below (animal 4516H, 20 pg/mL, animal 4519E, 180 pg/mL, and animal 4520E, 22 pg/mL) or close to the limit of quantification (animal 3515E, 345 pg/mL). After dosing, nine of 12 animals from the low-dose GNP group displayed levels above LLOQ on at least one occasion while all animals (12 of 12) in the high-dose group displayed levels above LLOQ on at least one occasion

On Day 28, all samples collected from placebo-control liquid and saline-control animals displayed undetectable levels of glucagon with the exception of two samples at 20 min post-dose (animals 1016D, 8644 pg/mL, and 2011D, 45 pg/mL). Those two values are probably due to variance of glucagon already produced in the body of the rats since those animals were both dosed before any treated animal; therefore, animals were deemed to have been dosed correctly. Following 28 consecutive daily dosings, all pre-dose samples collected from low- and high-dose groups were below or close to the limit of quantification indicating no accumulation, which is consistent with a fast systemic turnover of glucagon. After Day 28 dosing, nine of 12 animals from the low-dose group and all animals from the high-dose group displayed levels above LLOQ on at least one occasion. Mean AUC_0-t_ and C_max_ values were 43,036 pg.min/mL and 988 pg/mL for the low-dose group, respectively, and 60,073 pg.min/mL and 2109 pg/mL for the high-dose group, respectively (Fig. 3, Table 2). There were no statistical differences between sexes.

#### Hematology, coagulation, clinical chemistry, and urinalysis

There were no adverse test-article related changes in hematology, coagulation, clinical chemistry, and urinalysis parameters. A number of mean parameters differed from control values, with/without statistical significance, but the differences were independent of dose and sex or were minor in magnitude (Table 3). Thus, they were considered incidental and of no biological significance.

**Table 3:** Summary of hematology, coagulation, clinical chemistry, and urinalysis data in dogs, Day 29 relative to start date^a^

Parameter	Group 1: Placebo control		Group 2: Saline control		Group 3: Low dose		Group 4: High dose	
			Hematology					
	Male (*N* = 9)	Female (*N* = 10)	Male (*N* = 10)	Female (*N* = 10)	Male (*N* = 10)	Female (*N* = 9)	Male (*N* = 8)	Female (*N* = 9)
Red blood cells (10^12^/L)	8.62 (0.433)	8.08 (0.378)	8.52 (0.298)	8.14 (0.236)	8.59 (0.441)	8.19 (0.393)	8.49 (0.379)	8.26 (0.267)
Hemoglobin (g/L)	149 (6.8)	142 (6.2)	148 (5.7)	144 (3.1)	149 (6.7)	144 (6.4)	147 (7.7)	144 (3.7)
Hematocrit (L/L)	0.47 (0.019)	0.44 (0.015)	0.47 (0.019)	0.45 (0.010)	0.47 (0.023)	0.44 (0.021)	0.47 (0.027)	0.45 (0.011)
Mean corpuscular volume (fL)	55.1 (1.69)	54.5 (1.45)	55.4 (1.16)	55.1 (1.05)	55.0 (1.21)	54.3 (1.22)	55.1 (2.07)	54.7 (1.58)
Mean corpuscular hemoglobin (pg)	17.3 (0.35)	17.6 (0.52)	17.4 (0.25)	17.7 (0.41)	17.3 (0.37)	17.5 (0.42)	17.3 (0.71)	17.4 (0.40)
Mean corpuscular hemoglobin concentration (g/L)	314 (4.6)	323 (7.1)	314 (3.0)	321 (4.1)	315 (3.9)	323 (5.0)	313 (6.5)	319 (6.3)
Platelets (10^9^/L)	926 (148.3)	941 (105.1)	966 (186.9)	887 (123.7)	895 (169.0)	901 (136.8)	864 (122.1)	821 (194.5)
% Reticulocytes (%)	1.79 (0.652)	1.66 (0.517)	1.95 (0.477)	1.57 (0.381)	2.01 (0.377)	1.76 (0.463)	1.83 (0.320)	1.66 (0.457)
Reticulocytes (10^12^/L)	0.152 (0.0491)	0.133 (0.0379)	0.165 (0.0364)	0.128 (0.0318)	0.171 (0.0219)	0.143 (0.0363)	0.155 (0.0257)	0.136 (0.0363)
White blood cells (10^9^/L)	8.44 (1.498)	5.56 (1.867)	9.08 (1.476)	6.92 (1.547)	9.41 (2.417)	6.28 (2.144)	8.90 (2.046)	5.00 (1.472)
Neutrophils (10^9^/L)	0.74 (0.188)	0.40 (0.165)	0.82 (0.299)	0.39 (0.109)	0.85 (0.263)	0.47 (0.114)	0.82 (0.264)	0.43 (0.140)
Lymphocytes (10^9^/L)	7.40 (1.349)	4.94 (1.803)	7.89 (1.294)	6.21 (1.362)	8.20 (2.338)	5.59 (1.997)	7.73 (2.029)	4.37 (1.432)
Monocytes (10^9^/L)	0.13 (0.049)	0.11 (0.060)	0.19 (0.044)	0.16 (0.070)	0.18 (0.058)	0.10 (0.027)	0.19 (0.080)	0.09 (0.039)
Eosinophils (10^9^/L)	0.10 (0.026)	0.07 (0.023)	0.11 (0.040)	0.09 (0.034)	0.10 (0.033)	0.08 (0.017)	0.09 (0.027)	0.07 (0.033)
Basophils (10^9^/L)	0.03 (0.010)	0.02 (0.007)	0.03 (0.010)	0.02 (0.007)	0.03 (0.019)	0.02 (0.011)	0.03 (0.012)	0.02 (0.010)
Leucocytes (10^9^/L)	0.04 (0.012)	0.03 (0.015)	0.04 (0.017)	0.05 (0.039)	0.05 (0.033)	0.03 (0.027)	0.05 (0.022)	0.02 (0.011)
Coagulation								
	Male (*N* = 9)	Female (*N* = 10)	Male (*N* = 10)	Female (*N* = 10)	Male (*N* = 10)	Female (*N* = 9)	Male (*N* = 8)	Female (*N* = 9)
Prothrombin time (Sec)	13.5 (0.53)	13.3 (0.54)	13.4 (0.35)	13.7 (0.53)	13.3 (0.34)	13.5 (0.34)	13.6 (0.41)	13.7 (0.39)
Activated partial thromboplastin time (Sec)	17.1 (1.28)	15.5 (2.78) (*N* = 9)	16.5 (1.03)	16.6 (1.25)	16.8 (1.68)	16.2 (0.97)	16.1 (0.68)	16.2 (1.05)
Clinical chemistry								
	Male (*N* = 9)	Female (*N* = 10)	Male (*N* = 10)	Female (*N* = 10)	Male (*N* = 10)	Female (*N* = 10)	Male (*N* = 10)	Female (*N* = 10)
Alanine transaminase (U/L)	27 (4.5)	29 (8.7)	30 (5.4)	30 (7.4)	31 (8.7)	34 (14.3)	28 (7.1)	31 (9.9)
Aspartate aminotransferase (U/L)	138 (35.4)	114 (18.1)	124 (30.3)	120 (25.5)	148 (24.0)	135 (44.6)	130 (26.9)	120 (49.3)
Alkaline phosphatase (U/L)	140 (25.9)	70 (17.9)	139 (19.9)	70 (12.2)	135 (28.3)	65 (15.5)	127 (30.3)	55 (12.9)
Total bilirubin (μmolL)	2.1 (0.58) (*N* = 3)	1.9 (0.14) (*N* = 2)	2.0 (0.21) (*N* = 2)	1.9 (0.00) (*N* = 2)	1.9 (−) (*N* = 1)	2.1 (0.21) (*N* = 3)	2.2 (−) (*N* = 1)	2.2 (0.29) (*N* = 4)
Cholesterol (mmol/L)	1.80 (0.561)	1.85 (0.432)	1.67 (0.311)	1.84 (0.354)	1.49 (0.416)	2.0 (0.453)	1.76 (0.288)	1.75 (0.313)
Triglycerides (mmol/L)	0.57 (0.252)	0.28 (0.050)	0.57 (0.136)	0.36 (0.113)	0.55 (0.229)	0.38 (0.108)	0.57 (0.203)	0.34 (0.088)
Glucose (mmol/L)	7.5 (1.06)	6.9 (1.75)	7.1 (0.85)	8.5 (1.85)	7.4 (0.92)	8.1 (2.19)	8.4 (2.41)	9.1 (1.37)
Total protein (g/L)	59 (4.0)	61 (5.8)	59 (2.2)	62 (2.9)	59 (3.2)	64 (3.5)	61 (2.0)	65 (4.5)
Albumin (g/L)	39 (2.3)	44 (4.1)	39 (0.9)	43 (1.8)	38 (2.6)	45 (2.5)	39 (1.7)	48 (5.0)
Globulin (g/L)	20 (2.4)	17 (2.4)	21 (1.5)	19 (2.1)	21 (2.1)	19 (1.5)	22 (2.4)	18 (2.0)
Albumin/Globulin ratio	2.0 (0.24)	2.6 (0.30)	1.9 (0.12)	2.3 (0.26)	1.8 (0.23)	2.4 (0.17)	1.8 (0.24)	2.7 (0.53)
Urea (mmol/L)	5.0 (0.78)	5.4 (0.87)	4.6 (0.67)	5.2 (0.69)	5.1 (0.87)	5.8 (1.01)	5.6 (0.90)	5.1 (0.40)
Creatinine (μmolL)	22 (3.3)	28 (2.5)	21 (3.0)	28 (5.1)	25 (2.9)	28 (5.5)	26 (2.0)	29 (3.0)
Calcium (mmol/L)	2.55 (0.116)	2.52 (0.064)	2.60 (0.044)	2.57 (0.069)	2.59 (0.080)	2.63 (0.093)	2.60 (0.077)	2.68 (0.075)
Phosphate (mmol/L)	2.45 (0.162)	2.07 (0.126)	2.53 (0.145)	2.06 (0.249)	2.54 (0.174)	2.10 (0.224)	2.54 (0.269)	2.06 (0.213)
Sodium (mmol/L)	143 (1.1)	143 (1.7)	144 (1.5)	142 (1.8)	146 (1.0)	145 (2.1)	147 (1.4)	146 (1.4)
Potassium (mmol/L)	5.1 (0.20)	4.7 (0.39)	5.0 (0.31)	5.1 (0.35)	5.2 (0.32)	5.0 (0.47)	5.3 (0.60)	4.8 (0.26)
Chloride (mmol/L)	102 (1.9)	105 (1.9)	102 (1.6)	104 (2.3)	104 (0.9)	105 (1.6)	104 (1.7)	105 (1.3)
Urinalysis								
	Male (*N* = 9)	Female (*N* = 10)	Male (*N* = 10)	Female (*N* = 10)	Male (*N* = 10)	Female (*N* = 10)	Male (*N* = 10)	Female (*N* = 9)
Volume (mL)	7 (3.5)	7 (5.6)	9 (2.9)	5 (2.6)	7 (5.4)	5 (2.4)	10 (3.8)	9 (6.7)
Specific gravity	1.030 (0.0090)	1.028 (0.0138)	1.024 (0.0077)	1.027 (0.0083)	1.027 (0.0112)	1.026 (0.0079)	1.024 (0.0068)	1.021 (0.0156)

^a^All data are presented as mean (standard deviation)

#### Organ weights

There was a slight increase in organ weights relative to body weight in several organs (adrenals, brain, heart, kidneys, lungs/trachea, ovaries, and thyroid/parathyroid) in Group 1 female animals compared with all other groups. However, these differences were considered incidental and of no biological significance.

#### Macroscopic findings

Soft dark red material surrounding the heart and moderate, acute, multi-focal pulmonary intra-alveolar hemorrhage and many dark red areas in all lung lobes were observed in the placebo-control animal found dead (1006B). These findings, most likely due to physical trauma, contributed to the death of the animal. Since these observations were present in a placebo-control animal, they were considered incidental. Other observations were considered incidental or spontaneous. All gross pathology findings from all other main and recovery animals, regardless of experimental group, were considered spontaneous or incidental because of low incidence and inconsistency across control or treated groups.

#### Microscopic findings

Moderate, acute, multi-focal pulmonary intra-alveolar hemorrhage, mild mandibular lymph node sinusal congestion/hemorrhage, and mild, multi-focal acute thymic hemorrhage were observed in the placebo-control animal found dead (1006B). In comparison with the placebo-control and saline animals, GNP-related erosion/ulceration of the olfactory epithelium, frequently with minimal to mild acute to sub-acute inflammation of the lamina propria, was noted bilaterally or unilaterally at the dorsal turbinate of nasal cavity level 2, as a mild to moderate finding in 2/10 males and 3/10 females of Group 4 (high dose). Inflammation of the lamina propria of the olfactory epithelium was considered secondary to the erosion/ulceration of the olfactory epithelium. These lesions were always noted at the dorsal turbinate of the nasal cavity level 2, which suggested site-specificity of GNP-related insult in the nasal cavity. These lesions were not present in the recovery animals, suggesting reversibility of these lesions.

In Group 4 recovery animals, findings included retinal fold/rossette (1/10), cardiomyopathy (5/10), tubular basophilia of the kidneys (2/10), fibrosis of the kidneys (1/10), corticomedullary mineralization of the kidneys (5/10), healed infarct of the kidneys (1/10), focal cell infiltrate of the liver (1/10), focal necrosis of the liver (1/10), intra-alveolar hemorrhage of the lungs (3/10), increased cellularity of the mandibular lymph node (2/10), interstitial cell infiltrate of the prostate (1/5), inflammation of the prostate associated with urothelial hyperplasia (1/5), cell debris overlying epidermal hyperplasia (1/10), extramedullary hematopoiesis of the spleen (10/10), and hemorrhage of the thymus (1/10). The incidence and severity of these findings cannot be compared to control animals as there were no concurrent control saline recovery animals. However, these findings are common background lesions observed in Sprague–Dawley rats and were considered to be incidental or procedure-related rather than test-item related.

All other histopathology findings in other tissues, including those in placebo-control, saline, and low-dose groups were considered incidental or spontaneous or background and of no toxicological significance.

### Twenty-eight-day intra-nasal toxicity followed by a 14-day recovery period in beagle dogs

#### Formulation analysis

The mean glucagon content in the test-article devices on both Days 1 and 28 was 2.1 mg, therefore within 10 % of the desired value of 2 mg per device. The amount of glucagon on Days 1 and 28 was consistent; therefore, stability of glucagon throughout the 28 days at room temperature was confirmed. There was no glucagon present in any control article device on Day 1 or Day 28.

#### Efficiency of devices

Achieved powder concentrations of the devices are presented in Table 4.

**Table 4:** Achieved powder dose levels of GNP per animal

Group number	Group designation	Targeted powder dose (mg)	Achieved average powder dose (mg)	% efficiency
1	Placebo control	40.0	33.0^a^	82.5
			37.1^b^	92.8
3	Low dose	20.0	18.9^a^	94.5
			19.9^b^	99.5
4	High dose	40.0	36.8^a^	92.0
			39.6^b^	99.0

^a^Calculated using all values from the entire study including discharge weights obtained before implementation of a device wiping procedure to correct the adverse effect of device-related electrostatic charge on the analytical balance

^b^Calculated using values beginning April 8, 2011 (equivalent to study Day 10, 11, or 12 depending on the dog) after instituting a device wiping procedure to remove electrostatic charge and thus resulting in accurate and expected discharge weights

During the initial part of the study, the amounts of powder released from the devices appeared to be inconsistent, with several devices appearing to deliver less than expected and some devices apparently weighing more after discharge than before discharge. Following an investigation, these irregular values for test article discharge weights (i.e., delivery efficiency) were shown to be caused by an electrostatic charge on some of the devices that interfered with the device weight values obtained pre-dosing. The charge was removed by simply wiping each device with a small amount of alcohol. The actual amount given to the animals before the problem was discovered cannot be calculated accurately. However, since only a minimal amount of powder was present in devices that were opened after discharge, the animals were most likely given the correct dose; therefore, the impact on the study was assumed to be minimal. Also, as seen in Table 4, the placebo group was more affected by the electrostatic charge than the treated groups. As outlined in Table 4, the reported efficiency of the devices was between 82.5 and 94.5 % without wiping and between 92.8 and 99.5 % with wiping. However, efficiency values prior to implementation of corrective actions cannot be completely accurate since we cannot determine the exact amount discharged from the devices.

#### Mortality

There were no drug-related deaths in the study.

#### Clinical signs

With the exception of transient salivation and some sneezing in most dogs immediately after IN dosing with placebo powder or GNP, there were no treatment-related adverse clinical signs observed in this study. All clinical signs noted were considered incidental and not related to the administration of GNP since they were sporadic, infrequent, and/or present in control animals at a similar frequency and/or incidence.

#### Body weight

There were no body weight changes related to treatment with GNP. All body weight changes were considered incidental and not related to the administration of GNP.

#### Food consumption

Food consumption was unaffected by treatment with GNP.

#### Ophthalmoscopy

No treatment-related findings were noted during the course of this study and the observations recorded were incidental in origin and to be expected in this type of animal.

#### Electrocardiography

No treatment-related findings were noted on the electrocardiograms.

#### Toxicokinetics

**Fig. 4:**
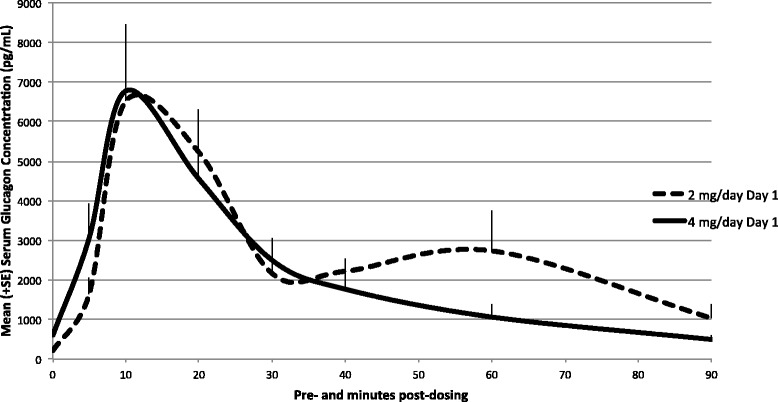
Mean (+ SE) uncorrected glucagon serum concentration profiles in dogs following a single intra-nasal administration—Day 1. Glucagon concentrations on Day 1 (linear scale). Levels were below the LLOQ (200 pg/mL) in all samples collected in the placebo-powder group on Day 1. Following a single GNP intra-nasal dose, mean glucagon concentration levels increased quickly to reach peak levels within 10 min and declined over the sampling interval. Mean peak glucagon levels were similar across GNP dose levels

Mean uncorrected glucagon concentration-time profiles of GNP on Day 1 are presented in Fig. 4. Glucagon concentration levels were below the LLOQ, 200 pg/mL, in all samples collected in the placebo-powder and saline-control groups on Day 1. Endogenous glucagon levels were observed in 11 of 12 dogs from GNP groups (animals 3502B and 3603B had reported values < LLOQ while animal 4001B had undetectable glucagon levels). Following a single GNP IN dose, mean glucagon concentration levels increased quickly in dog serum to reach peak levels within 10 min and declined over the sampling interval. Mean peak glucagon levels were similar across GNP dose levels.

**Fig. 5:**
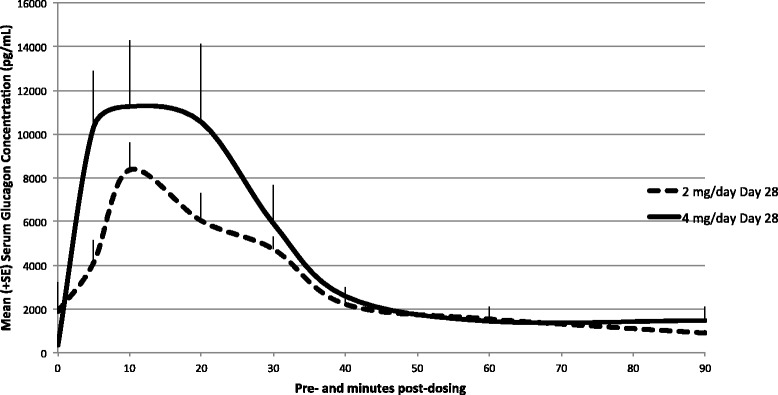
Mean (+ SE) uncorrected glucagon serum concentration profiles in dogs following multiple intra-nasal administrations—Day 28. Glucagon concentrations on Day 28 (linear scale). All samples collected from placebo-control powder and saline-control animals displayed glucagon levels < LLOQ with the exception of three samples, one from the saline-control group at pre-dose (animal 2503, 211 pg/mL) and three from the placebo-control powder group at pre-dose and 20 min post-dosing (animal 1501, 229 pg/mL, animal 1502, 355 pg/mL, and animal 1503, 201 pg/mL, respectively). On Day 28, following daily dosing for 28 consecutive days, pre-dose samples collected from low- and high-dose GNP groups generally displayed measurable glucagon levels

Mean uncorrected glucagon concentration-time profiles of GNP on Day 28 are presented in Fig. 5. On Day 28, all samples collected from placebo-control powder and saline-control animals displayed glucagon levels < LLOQ with the exception of four samples, one from the saline-control group at pre-dose (animal 2503, 211 pg/mL) and three from the placebo-control powder group at pre-dose and 20 min post-dosing (animal 1501, 229 pg/mL, animal 1502, 355 pg/mL, and animal 1503, 201 pg/mL, respectively). On Day 28, following daily dosing for 28 consecutive days, pre-dose samples collected from low- and high-dose GNP groups generally displayed measurable glucagon levels.

To comply with standard methodologies for endogenous compounds, a baseline correction was also applied to correct for endogenous glucagon levels and TK parameters were derived from corrected plasma concentration profiles. Baseline was defined as pre-dose values observed on Day 1. With baseline correction, after a single test-article administration on Day 1, the mean systemic exposures (AUC_0-t_) to glucagon were 213,385 and 148,561 pg.min/mL for the low- and high-dose groups, respectively. Median peak glucagon concentration was observed 10 min after dosing and increased with dose (6984 vs. 7251 pg/mL, for low- and high-dose groups, respectively). Following the last dose on Day 28, mean AUC_0-t_ (267,261 vs. 343,597 pg.min/mL for low- and high-dose groups, respectively) and C_max_ values (8684 vs. 13,171 pg/mL, for low- and high-dose groups, respectively) increased with dose (Table 5).

**Table 5:** Baseline-corrected toxicokinetic parameters of glucagon in dogs

Parameters	Mean (% CV)			
	Day 1		Day 28	
	GNP 2 mg/day	GNP 4 mg/day	GNP 2 mg/day	GNP 4 mg/day
N	6	6	6	6
AUC_0–90_ (pg min/mL)	229,465 (27.9)	151,619 (52.1)	267,261 (41.6)	345,747 (61.4)
AUC_0-t_ (pg min/mL)	213,385 (15.8)	148,561 (52.7)	267,261 (41.6)	343,597 (62.4)
C_max_ (pg/mL)	6984 (26.0)	7251 (50.6)	8684 (34.1)	13,171 (56.4)
T_max _^a^ (min)	10.0 (10.0, 20.0)	10.0 (5.00, 20.0)	10.0 (5.00, 10.0)	10.0 (5.00, 20.0)
T_1/2_ (min)	NC (NC)^b^	11.4 (NC)^c^	21.1 (18.4)^d^	7.79 (NC)^e^

*NC* not calculated

^a^Median (Min, Max); ^b^ *n* = 0; ^c^ *n* = 2; ^d^ *n* = 4; ^e^ *n* = 1

Although baseline glucagon levels were different between treatment groups, baseline levels were very low in both groups compared with glucagon levels observed post-dosing. These data suggest that animals treated with the experimental IN glucagon formulation experienced glucagon exposures well in excess of physiological levels. Consistent with the short half-life of glucagon in serum, minimal accumulation was observed following multiple GNP IN administrations and there were no statistical differences between sexes.

#### Hematology, coagulation, clinical chemistry, and urinalysis

There were no adverse test-article related changes in hematology, coagulation, clinical chemistry, and urinalysis parameters. No effect was seen on blood glucose as samples were taken prior to dosing with glucagon. Any increases or decreases observed were all within the reference range for that species and all differences from the control values, with/without statistical significance, were independent of dose and were minor in magnitude. Thus, they were considered to have no biological significance.

#### Organ weights

There were no prominent organ weight changes related to GNP-dosed animals and all differences from the control group weights were considered incidental and of no biological significance. A number of means differed from the control values, without statistical significance, but considering the inter-animal variability, they were regarded as incidental or of no biological significance.

#### Macroscopic findings

There were findings of a dark area and/or pale discoloration in the right middle lung lobe of one main animal in Group 2 and one main animal in Group 4, which was considered to be background or procedure-related following the microscopic evaluation. All other macroscopic findings were considered to be incidental or spontaneous because they were of low incidence or occurred in control and/or treated groups.

#### Microscopic findings

Minimal to moderate atrophy/degeneration of the olfactory epithelium (with/without sub-acute inflammation) was observed in level 4 of the nasal cavity of 2/6 (1/3 males and 1/3 females) Group 1 (placebo-control powder), 0/6 Group 2 (saline control), all Group 3 (low dose), and all Group 4 (high dose) animals. Since this finding was observed in Group 1, Group 3, and Group 4 animals, but not in Group 2, it was considered treatment-related. There was also an increase in the incidence and/or severity in the test-article treated animals in comparison to the Group 1 animals. Minimal atrophy/degeneration of the olfactory epithelium was also present in level 3 of the nasal cavity of one Group 4 male (4002B). Atrophy/degeneration of the olfactory epithelium was not observed in recovery animals, which suggests reversibility of the finding.

Minimal to moderate sub-acute bronchioloalveolar inflammation was observed in the lungs of 3/6 (2/3 males and 1/3 females) Group 1, 4/6 (3/3 males and 1/3 females) Group 2, all Group 3, and 4/6 (2/3 males and 2/3 females) Group 4 animals. This finding was observed in both control and treated animals and the mean severity grade did not vary in relationship to dose level of the test article, GNP. The sub-acute bronchioloalveolar inflammation of the lungs correlated with the macroscopic finding of dark and/or pale discoloration in one Group 2 and one Group 4 animal and was considered to be background or procedure-related.

Minimal to mild focal hyperplasia or squamous metaplasia of the respiratory epithelium was observed in the carina of 3/6 (2/3 males and 1/3 females) Group 1, 3/6 (2/3 males and 1/3 females) Group 2, 2/6 (0/3 males and 2/3 females) Group 3, and 4/6 (2/3 males and 2/3 females) Group 4 animals. Focal hyperplasia or squamous metaplasia of the respiratory epithelium was also present in one Group 1 animal and one Group 4 animal after the 14-day recovery period. This finding was observed in both the control and treated groups with an increase in incidence in Group 4 animals, but with no significant difference in the severity between groups. Hyperplasia and squamous metaplasia of the respiratory epithelium were regarded as adaptive responses following local irritation on the surface of the carinal epithelium and are not uncommon with inhalation of inert and/or non-toxic compounds.

All other microscopic findings were considered to be incidental, spontaneous, background, or procedure-related because they were of low incidence or occurred in control and/or treated groups.

### Single-dose intra-tracheal insufflation toxicity in rats

#### Dose administration

The individual amounts of GNP delivered to Group 2 animals are presented in Table 6. Following dosing accountability verification, the devices used for animals 2003B, 2005B, 2503B, and 2508D were determined to have had low efficiencies. The efficiencies were 46, 24, 58, and 58 %, respectively. Therefore, it was decided to replace these four animals with spare animals and dose them with new devices. The data from these four replacement animals (2103B, 2105B, 2603B, and 2608D) are included in Table 6.

**Table 6:** GNP doses administered per animal

Animal	Target (mg)	Actual (mg)	% of target
2001B	0.5	0.39	78
2002B	0.5	0.70	140
2103B	0.5	0.49	98
2004B	0.5	0.29	58
2105B	0.5	0.63	126
2006D	0.5	0.74	148
2007D	0.5	0.24	48
2008D	0.5	0.32	64
**Mean**	**0.5**	**0.48**	**95.0**
2501B	0.5	0.55	110
2502B	0.5	0.56	112
2603B	0.5	0.61	122
2504B	0.5	0.60	120
2505B	0.5	0.47	94
2506D	0.5	0.50	100
2507D	0.5	0.64	128
2608D	0.5	0.14	28
**Mean**	**0.5**	**0.51**	**101.8**

Mean values are shown in bold

#### Mortality

There were no deaths over the course of the study.

#### Clinical signs

There were no adverse clinical signs related to treatment with GNP. All clinical signs noted were considered incidental and not related to the administration of GNP.

#### Body weight

There were no body weight changes related to treatment with GNP.

#### Food consumption

There were no changes in food consumption that were considered related to treatment with GNP. All variations were considered of normal biological variation.

#### Organ weights

There were no changes in organ weights that were clearly related to treatment with GNP. Changes observed were sporadic and considered incidental and of normal variation.

#### Macroscopic findings

A dark area was observed in the lungs of 5/10 (4/5 males and 1/5 females) air-control (Group 1) rats and 2/10 (1/5 males and 1/5 females) GNP-treated (Group 2) rats at termination of the main study phase. The findings in the lungs and all other macroscopic findings were considered to be incidental, background, or procedure-related. These changes were not observed at termination of the recovery period.

#### Microscopic findings

Minimal alveolar histiocytosis and minimal alveolar hemorrhage were observed in the lungs of both air-control (Group 1) and GNP-treated (Group 2) rats. The alveolar histiocytosis was considered a normal non-specific pulmonary adaptive response associated with phagocytosis and clearance of inhaled particulates. These changes are commonly observed in inhalation studies, including from air-control animals, and could be related to inhalation of airborne particulates. The alveolar hemorrhage often correlated with the macroscopic finding of dark area and was considered to be procedure-related or background. The histiocytosis and alveolar hemorrhage were also present at the end of the 14-day recovery period. All other microscopic findings were considered to be incidental or spontaneous.

### Single-dose ocular tolerance in rabbits

#### Mortality

Three male rabbits (1001, 1102, and 1003) were treated with GNP. None of the test animals died during the course of this study.

#### Clinical signs

Clinical observations noted in the GNP-treated eye (left eye) and/or surrounding areas included one or more of the following: clear discharge, red conjunctiva, partial closure of the eye, and firm swelling of the periorbital area. These observations correlated with erythema and edema noted during ocular observations and grading, and were associated with possible mechanical irritation due to eyelid movements in presence of a solid powder in the eyes. All other clinical signs were considered incidental and are common in laboratory-housed members of this species.

#### Body weight

There was no body weight change related to the dosing administration of GNP. Body weights were measured for general health assessment, and showed expected progressive increases during the study period.

#### Ocular observations and grading

All animals presented slight unilateral (left eye) ocular findings related to the administration of GNP. In all cases, the findings noted at all time points (1, 24, 48, and 72 h) post-dosing, were limited to erythema (redness) and edema (swelling) of the conjunctiva and the palpebral membrane. These lesions were attributed to possible mechanical irritation due to eyelid movements in presence of solid powder in the eye since GNP was delivered to the eye as dry powder.

One animal (1001) presented a minimal area of fluorescein staining at 24 h post-treatment and none at the other time points. Ocular grading sheets only indicated a score of ‘1’ for the area of cornea involved with an opacity score of ‘0’. The score value of ‘1’ was intended for the area of ‘fluorescein staining’ following the observation after the fluorescein solution instillation for this animal. This was considered related to a trauma to the cornea during experimental procedures rather than a treatment-related effect.

#### Macroscopic findings

No GNP-related macroscopic changes were observed at necropsy. Animal 1102 presented a single, bilateral dark red area at the nictitating membrane of the eyes. This change was considered to be incidental and of no toxicological significance.

## Discussion

The objective of these studies was to evaluate in animal models the safety of GNP, a formulation of glucagon for intra-nasal administration intended for the treatment of severe hypoglycemia.

Intra-nasal administration of GNP for 28 consecutive days at the estimated average dose rate of up to 2 mg GNP/day (0.2 mg glucagon/day) was well tolerated by rats. There were two deaths during the study, but they were in the placebo-control group and a necropsy indicated the deaths were most likely due to physical trauma. There were no test-article related mortalities or adverse clinical signs, and body weights, food consumption, ophthalmoscopy findings, and clinical pathology parameters were unaffected by GNP. Adverse effects were limited to local effects observed at the site of administration at the high-dose only. Daily dosing over the course of 28 days revealed no evidence of accumulation of glucagon in the serum and there were no gender-related trends in glucagon exposure parameters. Both on Day 1 and Day 28, mean AUC_0-t_ and C_max_ values increased with dose. Daily dosing over the course of 28 days at the high-dose resulted in mild to moderate, unilateral or bilateral erosion/ulceration of the olfactory epithelium, frequently with minimal to mild acute to sub-acute inflammation of the lamina propria, at the dorsal turbinates of nasal cavity level 2, in 2/10 males and 3/10 females of Group 4 (0.2 mg glucagon/day). These GNP-related lesions showed complete resolution following the 14-day recovery period suggesting the adverse effects are reversible. The no-observed-effect level was considered to be at the low dose of 1 mg GNP/day (0.1 mg glucagon/day).

Dogs tolerated daily IN administration of GNP for 28 consecutive days at the estimated average dose up to 39.6 mg GNP/dog/day (4.0 mg glucagon/dog/day) without any mortality. With the exception of transient salivation and some sneezing in most dogs immediately after IN dosing, there were no treatment-related adverse clinical signs observed in this study. Body weights, food consumption, ophthalmoscopy, and electrocardiography data were unaffected by the treatment in any test-article treated animals. There were also no adverse test-article related changes in clinical pathology parameters. Daily dosing over the course of 28 days revealed minimal accumulation of glucagon in the serum and there were no gender-related trends in glucagon exposure parameters. With baseline correction, after a single test-article administration on Day 1, the mean systemic exposures (AUC_0-t_) to glucagon were 213,385 and 148,561 pg.min/mL for the low- and high-dose groups, respectively. Median peak glucagon concentration was observed 10 min after dosing and increased with dose (6984 vs. 7251 pg/mL, for low- and high-dose groups, respectively). Following the last dose on Day 28, mean AUC_0-t_ (267,261 vs. 343,597 pg.min/mL for low- and high-dose groups, respectively) and C_max_ values (8684 vs. 13,171 pg/mL, for low- and high-dose groups, respectively) increased with dose. Although baseline glucagon levels were different between treatment groups, baseline levels were very low in both groups compared with glucagon levels observed post-dosing. These data suggest that animals treated with the experimental IN glucagon formulation experienced glucagon exposures well in excess of physiological levels. There were no test-article related findings observed macroscopically. Microscopically, mild to moderate atrophy/degeneration of the olfactory epithelium was observed in the nasal cavity (level 4) of the Group 1 (placebo-control powder), Group 3 (low dose; 20 mg/day GNP [2.0 mg/day glucagon]), and Group 4 (high dose; 40 mg/day GNP [4.0 mg/day glucagon]) animals, with an increase of the incidence and severity in Group 3 and Group 4 animals. After a 14-day recovery period, this treatment-related finding in the nasal cavity was no longer present, suggesting reversibility of the finding.

A single intra-tracheal insufflation of GNP was well tolerated by rats. The estimated average doses administered were 0.48 and 0.51 mg GNP (0.04 and 0.05 mg glucagon) for males and females, respectively. There were no changes observed in any of the parameters evaluated in this study.

In the final study, male rabbits were treated with GNP by a single ocular instillation using a powder dosing device. There were no mortality or body weight changes following GNP administration during the course of this study. Clinical observations noted in the GNP-treated eye (left eye; treated with 30 mg GNP [i.e., 3 mg glucagon]) and/or surrounding areas included one or more of the following: clear discharge, red conjunctiva, partial closure of the eye, and firm swelling of the periorbital area. These observations correlated with erythema and edema noted during ocular observations and grading. At all ocular grading time points (1, 24, 48, and 72 h) post-dose, all animals presented slight unilateral (left eye) ocular findings related to the administration of GNP. In all cases, the findings were limited to erythema and edema of the conjunctiva and the palpebral membrane. These lesions were attributed to possible mechanical irritation due to eyelid movements in presence of solid powder in the eye since GNP was delivered to the eye as dry powder.

Glucagon has long been recognized for its wide safety margin. Eli Lilly reports the median lethal IV dose at 300 mg/kg for mice and 38.6 mg/kg for rats [10] while Novo Nordisk reports the LD_50_ (lethal dose, 50 %) following IV or SC injection in rats and mice to range from 100 to >200 mg/kg [11]. Daily administration of glucagon for 6 months to rats (1 mg/rat/day) or rabbits (1 mg/rabbit once or twice per day) did not cause adverse clinical signs, had no effect on growth or weight gain, and did not cause any pathological changes. The only treatment effect reported was an increase in liver glycogen content [12]. More recent data show daily IV injection of glucagon for 4 weeks to rats (0, 0.2, 1.0, and 5.0 mg/kg/day) and dogs (0, 1.0, and 5.0 mg/kg/day) was well tolerated [13]. There were no significant adverse clinical signs in rats or dogs. Body weight and food consumption were unaffected. Liver weight was increased in both species, but was not associated with any microscopic changes. There was no evidence of any effect on fertility after treatment of rats with up to 2 mg/kg BID with animal-derived glucagon [10]. There was no evidence of harm to the fetus after daily IV administration of 0.4, 2.0, or 10.0 mg/kg (i.e., 100–200 times greater than human exposure) of recombinant human glucagon to rats and rabbits during the period of fetal organogenesis [11].

Regarding IN administration, considerable data on cyclodextrins (CD), primarily on CDs that are much more water soluble than β-CD (i.e., randomly methylated β-CD, dimethyl β-CD, hydroxypropyl β-CD, α-CD), have been generated and reviewed [14]. Overall, the local safety profile of IN CDs appears to be very good, with results indicating local effects on nasal morphology. *In vitro* ciliary beat frequency and cytotoxicity profiles were generally similar to those observed with saline control and much less than those observed with bile salts or 0.01 % benzalkonium chloride [14]. More recently, a paper was published showing no evidence of tissue damage to rat nasal mucosa following 5 min of *in vivo* exposure to 5 and 20 % hydroxypropyl β-CD or to 1.5 % β-CD [15]. Testing of higher concentrations of β-CD was not possible because of β-CD’s very low water solubility, a factor that should contribute to both the local and systemic safety of intra-nasally administered β-CD.

The third ingredient, DPC, is not an approved excipient. DPC contains a choline group, a phosphate group, and a saturated aliphatic chain that is 12 carbons in length. All three moieties are present in phospholipids and lecithins ubiquitous in mammalian cell membranes. In addition, several *in vitro* and *in vivo* studies conducted by the authors have provided data that reveal no safety concerns pertaining to DPC (manuscript in development). Taken together, these results suggest that IN delivery of GNP holds promise as a future rescue medication for use by caregivers to treat insulin-induced hypoglycemic episodes in patients with type 1 or type 2 diabetes.

## Conclusions

In conclusion, IN administration of large overdoses of GNP to rats and dogs for 28 consecutive days was well tolerated and resulted in only mild to moderate fully reversible histological changes to nasal mucosa in some animals. Direct deposition into the lungs of rats did not result in any adverse findings, suggesting that inadvertent pulmonary exposure to GNP is likely to be well tolerated. In addition, a single ocular instillation using the intended powder dosing device pre-filled with 30 mg GNP to male New Zealand white rabbits was well tolerated, with minimal ocular irritation limited to slight erythema and edema localized to the conjunctiva and palpebral membrane.

## Additional file

Additional file 1:
**Manuscript supplement.** (DOCX 191 kb)

## Abbreviations

AAALAC: Association for Assessment and Accreditation of Laboratory Animal Care
ACC: Animal Care Committee
ADA: American Diabetes Association
AUC: Area under the curve
BID: *bis in die* (twice daily)
CD: Cyclodextrins
C_max_: Maximum concentration (in the plasma)
CI: Confidence intervals
CV: Coefficient of variation
DCE: Detailed clinical examination
DPC: Dodecylphosphocholine
ECG: Electrocardiogram
FDA: Food and Drug Administration
fL: Femtoliter
g: Gram
GNP: Glucagon nasal powder
IACUC: Institutional Animal Care and Use Committee
ID: Identification number
IN: Intranasal
IV: Intravenous
KIU: Kallikrein inhibitor unit (of aprotinin)
L: Litre
LD_50_: Median lethal dose
LLOQ: Lower limit of quantitation
LSM: Least square means
μmol: Micromole
mmol: Millimole
mL: Millilitre
NIH: National Institutes of Health
NRC: National Research Council
pg: Picogram
PR: PR interval (beginning of the P wave to beginning of QRS complex in an ECG)
QRS: RS complex in an ECG (represents rapid depolarization of right and left ventricles)
QT: QT interval in an ECG (beginning of the QRS complex to end of the T wave)
QTc: Corrected QT interval in an ECG
R_A_: Accumulation ratio
RPM: Revolutions per minute
SC: Subcutaneous
SD: Sprague–Dawley
SE: Standard error
Sec: Second
T_max_: Time for maximum plasma concentration of drug to be reached
TK: Toxicokinetic
U: Unit

## References

[1] Cryer PE (2002). Hypoglycaemia: the limiting factor in the glycaemic management of Type I and Type II Diabetes. Diabetologia.

[2] Deary IJ, Frier BM, Fisher M (2007). Symptoms of hypoglycaemia and effects on mental performance and emotions. Hypoglycaemia in clinical diabetes.

[3] Cryer PE, Davis SN, Shamoon H (2003). Hypoglycemia in diabetes. Diabetes Care.

[4] Leckie AM, Graham MK, Grant JB, Ritchie PJ, Frier BM (2005). Frequency, severity, and morbidity of hypoglycemia occurring in the workplace in people with insulin-treated diabetes. Diabetes Care.

[5] Zammitt NN, Frier BM (2005). Hypoglycemia in type 2 diabetes: pathophysiology, frequency, and effects of different treatment modalities. Diabetes Care.

[6] UK Hypoglycemia Study Group (2007). Risk of hypoglycaemia in types 1 and 2 diabetes: effects of treatment modalities and their duration. Diabetologia.

[7] Cryer PE (2009). The clinical problem of hypoglycemia in diabetes. Hypoglycemia in diabetes: pathophysiology, prevalence, and prevention.

[8] American Diabetes Association (2015). Standards of medical care in diabetes—2015. Diabetes Care.

[9] Jain MK (1988). Components of biological membranes. Introduction to biological membranes.

[10] GLUCACON (glucagon for injection, rDNA origin) Product Monograph. Toronto, Ontario: Eli Lilly Canada Inc.; 2012 http://www.lilly.ca/en/pdf/product-monograph/04_rglucagon-pm_9july2012.pdf. Accessed 26 September 2015.

[11] GLUCAGEN® and GLUCAGEN® Hypokit 1 mg (glucagon) Product Monograph. Mississauga, Ontario: Novo Nordisk Canada Inc.; 2014. http://www.paladin-labs.com/our_products/PM_GlucaGen_EN.pdf. Accessed 26 September 2015.

[12] Root MA, Ellis J, Staub A (1954). Effect of glucagon on insulin hypoglycemia. Proc Soc Exp Biol Med.

[13] Eistrup C, Hjortkjaer RK, Pickersgill N, Virgo DM, Woolley AP (1993). Glucagon produced by recombinant DNA technology: repeated dose toxicity studies, intravenous administration to CD rats and beagle dogs for four weeks. Pharmacol Toxicol.

[14] Merkus FW, Verhoef JC, Marttin E, Romeijn SG, van der Kuy PH, Hermens WA (1999). Cyclodextrins in nasal drug delivery. Adv Drug Deliv Rev.

[15] Asai K, Morishita M, Katsuta H, Hosoda S, Shinomiya K, Noro M (2002). The effects of water-soluble cyclodextrins on the histological integrity of the rat nasal mucosa. Int J Pharm.

