# Supplementary material for: A novel nasal powder formulation of glucagon: toxicology studies in animal models
Source: BMC Pharmacol Toxicol. 2015 Oct 26;16:29. doi: 10.1186/s40360-015-0026-9 (PMC4621930; doi:10.1186/s40360-015-0026-9)
Supplement: Additional file 1: — Manuscript supplement. (DOCX 191 kb) [file 40360_2015_26_MOESM1_ESM.docx]

**Supplementary Information**

**A novel nasal powder formulation of glucagon: toxicology studies in animal models**

Frederick E. Reno^1^, Patrick Normand^2^, Kevin McInally^2^, Sherwin Silo^3^, Patricia Stotland^4^, Myriam Triest^4^, Dolores Carballo^4^, Claude Piché^4*^

^1^130 Macaw Lane, Merritt Island, FL 32952, USA

^2^ITR Laboratories Canada Inc. (ITR), 19601 Clark Graham Blvd, Baie d’Urfe, Quebec, Canada

^3^ CiToxLAB North America, 445 Armand-Frappier Blvd, Laval (Québec), Canada

^4^ Locemia Solutions ULC, 8505 Dalton, Montreal, QC, Canada

^*^Correspondence: [claude.piche@locemia.com](mailto:claude.piche@locemia.com)

E-mail addresses:

FR: [freno1@cfl.rr.com](mailto:freno1@cfl.rr.com)

PN: [pnormand@itrlab.com](mailto:pnormand@itrlab.com)

KM: [kmcinally@itrlab.com](mailto:kmcinally@itrlab.com)

SS: [silos@ca.citoxlab.com](mailto:silos@ca.citoxlab.com)

PS: [patricia.stotland@locemia.com](mailto:patricia.stotland@locemia.com)

MT: [myriam.triest@locemia.com](mailto:myriam.triest@locemia.com)

DC: [dolores.carballo@locemia.com](mailto:dolores.carballo@locemia.com)

CP: [claude.piche@locemia.com](mailto:claude.piche@locemia.com)

**28-Day Intra-nasal Toxicity Followed by a 14-Day Recovery Period in Rats**

**Test System**

Species: Rat (*Rattus norvegicus*)

Strain: Sprague-Dawley Crl:CD (SD)

Source: Charles River Canada Inc., 188 rue Lasalle, St-Constant, Quebec, Canada

Total Animal No. in Study: 142 (71 males, 71 females) and 14 spares

Body Weight Range: 281-393 g for males and 210-286 g for females at onset of treatment

Age Range: 9-10 weeks at onset of treatment

Acclimation Period: 2 weeks minimum

Allocation to Study Groups

During the acclimation period, 71 male and 71 female rats were assigned to their respective dose groups by block randomization based on body weights.

Animal Replacement

During the acclimation period, animal 3009B was replaced by a spare animal from the same shipment and maintained under the same environmental conditions due to the presence of a small lump on the abdomen. The replacement animal new ID number was 3109B.

Administration of the Test and Control/Vehicle Articles

The expected glucagon dosages per rat are indicated in the table below:

| Group Number | Group Designation | Dose Level of  Glucagon (mg/day) | Volume administered  (µL) |
| --- | --- | --- | --- |
| 1 | Placebo control | 0 | 16/nostril |
| 2 | Control saline | 0 | 16/nostril |
| 3 | Low dose | 0.1 | 8/nostril |
| 4 | High dose | 0.2 | 16/nostril |

Test article accountability was taken for all groups. Groups 3 and 4 accountability was done together as there was only one tube for both groups.

All test and control articles were administered using a micropipette, while changing micropipette tip on each administration, to instill the dose in the external nares. With the animal suitably restrained and the head held such that the nose is tipped up slightly above the horizontal plane, the pipette was brought as close as possible to the external nares and the liquid was deposited. 16 µL was deposited in each nostril for Groups 1, 2, and 4, and 8 µL was deposited in each nostril for Group 3. The test article formulations (Groups 1, 3, and 4) were prepared daily just prior to dosing.

**In-life Observations**

Except where stated otherwise, data recorded for the animals in the toxicokinetic (TK) groups were limited to mortality, cage-side clinical signs, and body weight. These data are maintained on file at ITR but are not reported. For toxicology group animals, only the data collected during the 1-week period immediately prior to treatment were reported for the pre-treatment period. Additional data are maintained with the raw data for the study.

Mortality

Mortality checks were performed at least once per day during all phases of the study. Animals that died prematurely were subjected to detailed external and internal necropsy examination. Tissues from these animals were collected and preserved according to the Tissue Preservation section of this protocol.

Clinical Observations

For all main and recovery animals, cage-side clinical signs (ill health, behavioral changes, etc.) were recorded at least once daily during the acclimation period and at least once per day (am) during the treatment and recovery periods, except on detailed clinical examination (DCE) days. DCE was performed at least once pre-treatment, weekly during the treatment and recovery periods, and before necropsy. For TK animals, cage-side observations were recorded manually throughout the study only when abnormal clinical signs were observed. No DCE was performed on these animals.

Body Weights

Body weights were recorded for all animals at least once prior to group assignment and approximately 1 week prior to initiation of treatment. Body weights were recorded for all animals up to 1 day prior to dosing and at least once weekly thereafter during the treatment (at the same time period prior to dosing on each occasion) and recovery periods, as well as terminally prior to necropsy (fasted).

Food Consumption

Individual weekly food intake was recorded for all main and recovery animals during the last week of the pre-treatment period and throughout the treatment and recovery periods. For recovery animals, during the last week of treatment, food consumption followed the feeding regimen of the main animals (i.e., 6 days of consumption only recorded). For these animals the food consumption period restarted immediately and continued on a weekly basis to the end of the recovery period.

Ophthalmoscopy

Fundoscopic (indirect ophthalmoscopy) and biomicroscopic (slit lamp) examinations were performed once for all animals during the pre-treatment period and once for all main and recovery animals during Week 4 of the treatment period.

Toxicokinetics

A series of 7 blood samples (approximately 0.5 mL each) were collected from Groups 1, 3, and 4 TK phase rats on each of Days 1 and 28 of the treatment period. On each occasion, samples were collected prior to dosing, and at 10, 20, 30, 40, 60, and 90 min after treatment. This regimen allowed each rat to be sampled on three or four occasions with a total blood volume not exceeding 2.0 mL removed on each TK bleeding day. One blood sample (approximately 0.5 mL each) was collected from Group 2 TK phase rats on each of Days 1 and 28 of the treatment period. On each occasion, samples were collected at 20 min after treatment. This regimen allowed each Group 2 rat to be sampled on one occasion with a total blood volume of 0.5 mL removed on each TK bleeding day. For this purpose, each rat (unanesthetized) was bled by jugular venipuncture and the samples were collected into tubes without anti-coagulant containing 250 KIU of aprotinin per mL of whole blood. Following its last blood sampling, each animal was euthanized by CO_2_ asphyxiation followed by cervical dislocation and was discarded without further examination. In-life observations (mortality and clinical signs [when present]) for these animals were recorded but are not reported.

| Group  Number | Number of  Animals/Sex | Toxicokinetic Time Point (min post-dose) | | | | | | |
| --- | --- | --- | --- | --- | --- | --- | --- | --- |
|  |  | pre | 10 | 20 | 30 | 40 | 60 | 90 |
| 1 | 3 | √ |  | √ |  | √ |  | √ |
|  | 3 |  | √ |  | √ |  | √ |  |
| 2 | 3 |  |  | √ |  |  |  |  |
| 3 | 3 | √ |  | √ |  | √ |  | √ |
|  | 3 |  | √ |  | √ |  | √ |  |
| 4 | 3 | √ |  | √ |  | √ |  | √ |
|  | 3 |  | √ |  | √ |  | √ |  |

Blood was collected into tubes without anti-coagulant containing 250 KIU of aprotinin per mL of whole blood and allowed to clot for 20-30 min at room temperature. Aprotinin was added because glucagon must be protected from proteolysis during assay procedures and sample storage. Procedures for adding aprotinin into the tubes are in the raw data. The samples were then centrifuged (at ~2700 RPM for 10 min) and the resulting serum was recovered, aliquoted into vials each containing up to 125 μL, and stored frozen (approximately -80°C nominal) in labeled vials or tubes until shipment (on dry ice) to the bioanalytical laboratory.

Deviations to the TK time points were noted in the raw data and were made available with the samples. The location of blood withdrawal was noted in the raw data.

All blood samples were sent to the bioanalytical laboratory; however, only the following samples were analyzed:

- Group 1: Pre-treatment and 20 min post-treatment
- Group 2, 3, and 4: All samples collected were analyzed

The stability of the test article in the biological matrix was demonstrated according to accepted bioanalytical assay validation requirements to cover the duration from sample collection until completion of sample analysis and at the storage conditions used in the study.

**Clinical Pathology**

Blood/Urine Sampling

Laboratory investigations (hematology, coagulation, clinical chemistry, and urinalysis) were performed on all main and recovery animals at termination.

Blood samples were collected from the abdominal aorta or by cardiac puncture at termination. Urine was collected (over approximately 16 to 18 h) by placing a collection tray under the rats’ home cages or by placing them in metabolism cages. Animals were deprived of food during these collections, except for recovery animal 4513G, which was not starved overnight. However, urinalysis parameter values did not differ from comparable recovery animals from the same group. For urine collections at the end of the study, the urine collection time and associated food deprivation was extended to 23 h for animal 4501B since no urine had been collected during the 16- to 18-h period.

Hematology, Coagulation, Clinical Chemistry, and Urinalysis

Standard parameters were measured for the criteria of hematology, coagulation, clinical chemistry, and urinalysis.

**Necropsy Procedures**

Gross Examination and Organ Weights

The main and recovery animals were euthanized upon completion of the treatment/observation periods and following an overnight period without food. These animals were anesthetized with isoflurane, to allow collection of blood samples for clinical pathology evaluation, followed by exsanguination.

To avoid autolytic changes, the necropsy examination of the carcass was conducted as soon as possible, on all animals that died or were euthanized at the study conclusion. Gross pathology consisted of an external examination, including identification of all clinically recorded lesions, as well as a detailed internal examination. For main and recovery animals euthanized at termination, organs identified in the Tissue Preservation section were dissected, trimmed free of fat, and weighed. Body weight-relative organ weights were calculated.

Tissue Preservation

On completion of the gross pathology examination and selected organ weighing, the tissues and organs noted below were retained. Neutral buffered 10% formalin was used for fixation and preservation unless otherwise indicated.

| **ORGANS/TISSUES** | | **Retained**  **(**•**)** | **Weighed**  **( )** | **Examined**  **(**€**)** | **ORGANS/TISSUES** | **Retained**  **(**•**)** | **Weighed**  **( )** | **Examined**  **(**€**)** |
| --- | --- | --- | --- | --- | --- | --- | --- | --- |
| Adrenals | | • | 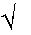 | € | Duodenum | • |  | € |
| Animal identification | | • |  |  | Jejunum | • |  | € |
| Aorta (thoracic) | | • |  | € | Ileum | • |  | € |
| Blood | | • |  |  | SC, cervical | • |  | € |
| Bone marrow smears (3) | | • |  |  | Spleen | • | 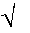 | € |
| Brain | | • | 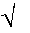 | € | Sternum and marrow | • |  | € |
| Cecum | | • |  | € | Stomach | • |  | € |
| Colon | | • |  | € | Testes | •d | 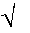 | € |
| Epididymides | | •d |  | € | Thymus | • | 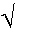 | € |
| Esophagus | | • |  | € | Thyroid gland/parathyroids | • | 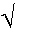 | € |
| Eyes | | •a |  | € | Tongue | • |  | € |
| Femur and marrow | | • |  | € | Trachea | •c |  | € |
| Heart | | • | 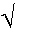 | € | Urinary bladder | • |  | € |
| Kidneys | | • | 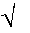 | € | Uterus | • | 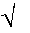 | € |
| Liver (2 lobes) | | • | 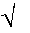 | € | Vagina | • |  | € |
| Lungs (2 lobes) | | •bc | 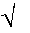 | € |  |  |  |  |
| LN, mandibular | | • |  | € | Abnormal findings | • |  | € |
| LN, mesenteric | | • |  | € |  |  |  |  |
| Mammary gland (inguinal) | | • |  | € |  |  |  |  |
| Optic nerves | | •a |  | € | **Additional tissues presented below** | | | |
| Ovaries | | • | 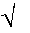 | € |  |  |  |  |
| Pancreas | | • |  | € | Nasopharynx | • |  | € |
| Pituitary | | • | 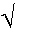 | € | Nasal cavity | • |  | € |
| Prostate | | • | 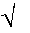 | € |  |  |  |  |
| Rectum | | • |  | € |  |  |  |  |
| SG, mandibular | | • |  | € |  |  |  |  |
| Sciatic nerve | | • |  | € |  |  |  |  |
| Seminal vesicles | | • |  | € |  |  |  |  |
| Skeletal muscle | | • |  | € |  |  |  |  |
| Skin and subcutis (inguinal) | | • |  | € |  |  |  |  |
|  | | | | | | | | |
| a | Davidson’s fluid (euthanized animal only) | | | | | | | |
| b | Lungs were infused with 10% neutral buffered formalin (euthanized animal only) | | | | | | | |
| c | Lungs were weighed with trachea and recorded in Provantis as lungs in organ weight record | | | | | | | |
| d | Bouin’s fluid (euthanized animal only) | | | | | | | |
| LN | Lymph node | | | | | | | |
| SG | Salivary gland | | | | | | | |
| SC | Spinal cord | | | | | | | |
| € | Examined microscopically | | | | | | | |
|  |  | | | | | | | |
| **Notes:** | | | | | | | | |
| Paired organs weighed together.  Parathyroids, mammary gland, and optic nerves were only examined histologically if present in routine sections. | | | | | | | | |

**Histopathology Procedures**

Slide Preparation

Tissues, detailed in the Tissue Preservation section, were prepared for microscopic examination by embedding in paraffin wax, sectioning, and staining with hematoxylin and eosin. Histological processing was conducted for all tissues detailed in the Tissue Preservation section for animals detailed below.

Histopathological Examination

Histopathological examination was performed on:

1. Esophagus, lungs, nasal cavity, nasopharynx, duodenum, ileum, jejunum, stomach, and trachea from animals found dead
2. Esophagus, lungs, duodenum, ileum, jejunum, stomach, and trachea from all main and recovery Group 1 animals
3. All gross lesions from all animals, except for abnormal liver finding for animal 3502F as it was not available for histopathological examination. However, there were no findings in the liver for the other animals, therefore it does not impact the study
4. Nasal cavity and nasopharynx tissues from all animals
5. All tissues depicted in the tissue preservation table for all Group 2 and 4 animals

Pituitary of animal 4013G, sciatic nerve of animal 4014G, and urinary bladder of animals 2508B and 4515G were not available for histopathological examination; however, there were no findings in these tissues for the other animals of Groups 2 and 4; therefore, it does not affect the pathological interpretation.

**Toxicokinetic Analysis**

TK analysis was performed in WinNonlin Enterprise v5.2 [1]. All tables and figures were completed using Microsoft Office (Excel) 2003 [2], SigmaPlot v9.0.1 [3], and WinNonlin AutoPilot [4] v1.1.1.

Non-compartmental analysis (NCA) was performed using model 200 (Extravascular Input) in WinNonlin, with the sparse sampling module. Briefly, the sparse sampling module uses the individual animal information to calculate standard errors that will account for any correlations in the data resulting from repeated sampling of individual animals. A standard error was calculated for the mean area under the curve from dosing time through the time of last quantifiable concentration (AUC_0-t_) and for the maximum concentration value (C_max_). Standard error of the mean AUC was calculated as described by Nedelman and Jia [5] using a modification reported by Holder [6]. Standard error of the mean C_max_ was calculated as the sample standard deviation of the y-values at time t_max_ divided by the square root of the number of observations at t_max_, or equivalently, the sample standard error of the y-values at t_max_.

**Statistical Analyses**

Descriptive statistics

Descriptive statistics (N, mean, standard error [SE], minimum, median, maximum, and percent coefficient of variation [CV%] for concentrations) were calculated using WinNonlin AutoPilot, or Excel and reported as appropriate (minimal sample size of 1 for mean, minimum, median, maximum, and variance derived statistics). Data are presented with statistical validity, with the spread of data determining the significant figures for reporting of data. Values over 100 were reported to the nearest integer, when possible. CV% was reported to one decimal. All figures were presented as mean ± SE.

# 2. 28-Day Intra-nasal Toxicity Followed by a 14-Day Recovery Period in Beagle Dogs

**Test System**

Species: Dog (*Canis familiaris)*

Strain: Beagle

Source: Marshall BioResources, Inc., North Rose, New York, 14516 USA

Total Animal No. in Study: 32 (16 males, 16 females) and 4 spares

Body Weight Range: 6.5-8.9 kg for males and 6.7-7.9 kg for females at onset of treatment

Age Range at Start: 6-7 months old at onset of treatment

Acclimation Period: Approximately 2 weeks

Allocation to Study Groups

During the acclimation period, each of 16 male and 16 female dogs was assigned to the appropriate dose group by a process of block randomization based on body weight.

Animal Replacement

Animal 3503B was replaced by a spare animal from the same shipment and maintained under the same environmental conditions due to ophthalmology rejection. Replacement animal new ID number was 3603B.

Administration of the Test and Control Articles

The expected glucagon dosages per dog are indicated in table below:

| **Group Number** | **Group Designation** | **Targeted Dose of Glucagon**  **(mg/day)** |
| --- | --- | --- |
| 1 | Placebo-control powder | 0 |
| 2 | Saline control | 0 |
| 3 | Low dose | 2.0 |
| 4 | High dose | 4.0 |

Placebo-control powder and glucagon nasal powder (GNP, low- and high-dose) articles were administered by intra-nasal injection, while the dogs were restrained on a sling, using a powder delivery device (UnitDose Powder, Aptar Pharma) provided by the sponsor. To estimate the actual amount of drug delivered to each dog, all test and placebo-control delivery devices were weighed before and after administration of the powder on an analytical scale. Each device contained 20 mg powder.

For dogs in the low-dose group, the entire dose was administered in one nostril from a single device. Therefore, each dog received 20 mg powder per day with a total of 2 mg glucagon. Alternate nostrils were used for each dosing episode for this group of animals. For example, these dogs were dosed on Day 1 using the left nostril; for the next day, the right nostril was used and *vice versa* for the balance of the study.

For dogs in the high-dose group, one device was discharged into each nostril each day. Therefore, each dog received 20 mg powder per day per nostril with a total of 4 mg glucagon.

For dogs in the placebo-control powder group, the dose consisted of one device per nostril per dog per day with each dog receiving 40 mg placebo powder per day.

For dogs in the saline-control group, a micropipette was used to deliver 10 µL of saline in each nostril (i.e., 20 µL total) of each dog every day during the dosing period.

**In-life Observations**

Mortality

Mortality checks were performed at least once per day during all phases of the study.

Clinical Observations

Cage-side clinical signs (ill health, behavioral changes, etc.) were recorded at least once daily during the acclimation period and at least once per day (am) during the treatment and recovery periods except on DCE days, where the morning cage-side clinical signs were replaced by a DCE. A DCE of each dog was performed at least once pre-treatment and before necropsy**.**

Body Weights

Body weights were recorded for all animals at least once prior to group assignment and approximately 1 week prior to initiation of treatment. Body weights were recorded for all animals up to 1 day prior to dosing and at least once weekly thereafter during the treatment (at the same time period before dosing) and recovery periods, as well as terminally prior to necropsy (fasted).

Food Consumption

Individual daily food intake was recorded for all animals during the last week of the pre-treatment period and daily throughout the treatment and recovery periods.

Ophthalmoscopy

Fundoscopic (indirect ophthalmoscopy) and biomicroscopic (slit lamp) examinations were performed for all animals, once during the pre-treatment period and once during Week 4 of the treatment period.

Electrocardiography

Electrocardiograms (ECGs, limb leads I, II, and III, and augmented leads aVR, aVL, and aVF) were obtained for all dogs once during the pre-treatment period and during Week 4 of the treatment period.

The tracings were assessed for gross changes indicative of cardiac electrical dysfunction and the potential presence of abnormalities involving heart rate (lead II), sinus and atrioventricular rhythm, or conductivity were determined. Heart rate, PR interval, QRS duration, QT, and QTc interval values were tabulated for incorporation into the study report. A sling was utilized to restrain each animal during the recording of its ECG.

Toxicokinetics

A series of 10 blood samples (approximately 1.0 mL each) was removed from each main dog on each of Days 1 and 28 of the treatment period. For this purpose, each dog was bled by venipuncture and the samples were collected into tubes without anti-coagulant containing 250 KIU of aprotinin per mL of whole blood. On each occasion, samples were collected at 30 min, 15 min, and immediately prior to dosing and 5, 10, 20, 30, 40, 60, and 90 min after treatment. The animals were restrained on a sling and an Abbocath (or “indwelling catheter”) was placed in an appropriate vein where required for blood sample collection. However, blood samples taken 90 min after treatment were taken from the jugular vein without the animal on the sling.

Blood was collected into tubes without anti-coagulant containing 250 KIU of aprotinin per mL of whole blood and allowed to clot for 20-30 min at room temperature. Aprotinin was added because glucagon must be protected from proteolysis during assay procedures and sample storage. The samples were centrifuged (at ~2700RPM for 10 min) and the resulting serum was recovered, aliquoted into vials each containing up to 125 μL, and stored frozen (approximately -80°C nominal) in labeled vials or tubes until shipment (on dry ice) to the bioanalytical laboratory.

Deviations from the TK time points were noted in the raw data and were made available with the samples. The location of blood withdrawal was noted in the raw data. All blood samples were sent to the bioanalytical laboratory; however, only the following samples were analyzed:

- Groups 1 and 2: Immediately prior to dosing and 20 min post-dosing
- Groups 3 and 4: Immediately prior to dosing, and 5, 10, 20, 30, 40, 60, and 90 min

post-dosing

The stability of the test article in the biological matrix, except for the long-term stability assessment at -80^o^C, which is still ongoing and will be completed after finalization of this report, was demonstrated according to accepted bioanalytical assay validation requirements to cover the duration from sample collection until completion of sample analysis and at the storage conditions used in the study. These data were confirmed in the TK report or in appropriate documentation. However, TK results will only be confirmed after completion of long-term stability assessment and documentation for confirmation of the long-term stability will be provided to and kept with the sponsor upon completion.

Clinical Pathology Blood/Urine Sampling

Laboratory investigations (hematology, coagulation, clinical chemistry, and urinalysis) were performed on all animals prior to start of treatment, on Day 28, and at the end of the recovery period. Coagulation samples for the recovery animals were stored at -20^o^C for 12 days before laboratory investigations were performed instead of being stored at -80^o^C for a maximum of 7 days. However, there were no changes in the coagulation parameters between pre-treatment and Day 28, and values were within normal limits. Therefore, coagulation parameter results for recovery animals are considered valid.

Blood samples were collected by venipuncture following an overnight period of food deprivation consisting of at least 12 h. Urine was collected from animals deprived of food and water, overnight (at least 16 h, but no more than 20 h for water).

Various clinical pathology parameters are automatically recorded by the instrumentation, but these are not reported. Residual samples of plasma or serum will be considered expired as of 3 months after collection, and will be disposed of thereafter, in accordance with ITR Standard Operating Procedures. Blood from hematology and urine from urinalysis samples were considered expired as of 24 h after analysis, and were disposed of thereafter, in accordance with ITR Standard Operating Procedures.

Hematology, Coagulation, Clinical Chemistry, and Urinalysis

Standard parameters were measured for the criteria of hematology, coagulation, clinical chemistry, and urinalysis.

**Necropsy Procedures**

Gross Examination and Organ Weights

All animals were euthanized upon completion of the treatment/recovery periods and following an overnight period without food. The dogs were pre-anesthetized with acepromazine and then euthanized by an intravenous overdose of sodium pentobarbital followed by exsanguination by transection of major blood vessels. For each dog, the necropsy consisted of an external examination, including reference to all clinically recorded lesions, as well as a detailed internal examination. A staff pathologist was available for consultation during all necropsies.

To avoid autolytic changes, the necropsy examination of the carcass was conducted as soon as possible, on all animals euthanized at the conclusion of the study. Organs identified in the Tissue Preservation section were dissected, trimmed free of fat, and weighed. Body weight relative organ weights were calculated.

Tissue Preservation

On completion of the gross examination and selected organ weighing, the tissues and organs noted below were retained. Neutral buffered 10% formalin was used for fixation and preservation unless otherwise indicated.

| **ORGANS/TISSUES** | | **Retained**  **(**•**)** | **Weighed**  **(**√**)** | **Examined**  **(**€**)** |  | **ORGANS/TISSUES** | **Retained**  **(**•**)** | **Weighed**  **(**√**)** | **Examined**  **(**€**)** |
| --- | --- | --- | --- | --- | --- | --- | --- | --- | --- |
| Adrenals | | • | √ | € |  | Sciatic nerve | • |  | € |
| Animal identification | | • |  |  |  | Skeletal muscle | • |  | € |
| Aorta (thoracic) | | • |  | € |  | Skin and subcutis (inguinal) | • |  | € |
| Blood | |  |  |  |  | Duodenum | • |  | € |
| Bone marrow smears (3) | | • |  |  |  | Jejunum | • |  | € |
| Brain | | • | √ | € |  | Ileum | • |  | € |
| Cecum | | • |  | € |  | SC, cervical | • |  | € |
| Colon | | • |  | € |  | Spleen | • | √ | € |
| Epididymides | | •d |  | € |  | Sternum and marrow | • |  | € |
| Esophagus | | • |  | € |  | Stomach | • |  | € |
| Eyes | | •a |  | € |  | Testes | •d | √ | € |
| Femur and marrow | | • |  | € |  | Thymus | • | √ | € |
| Gallbladder | | • |  | € |  | Thyroid gland/parathyroids | • | √ | € |
| Heart | | • | √ | € |  | Tongue | • |  | € |
| Kidneys | | • | √ | € |  | Trachea | •c |  | € |
| Liver (2 lobes) | | • | √ | € |  | Urinary bladder | • |  | € |
| Lungs (all lobes) | | •b | √c | € |  | Uterus | • | √ | € |
| LN, mandibular | | • |  | € |  | Vagina | • |  | € |
| LN, mesenteric | | • |  | € |  |  |  |  |  |
| Mammary gland (inguinal) | | • |  | € |  | Abnormal findings | • |  | € |
| Optic nerves | | •a |  | € |  |  |  |  |  |
| Ovaries | | • | √ | € |  |  |  |  |  |
| Pancreas | | • |  | € |  |  |  |  |  |
| Pituitary | | • | √ | € |  | **Additional Tissues presented below** |  |  |  |
| Prostate | | • | √ | € |  | Nasal cavity (all 4 levels) | • |  | € |
| Rectum | | • |  | € |  | Nasopharynx | • |  | € |
| SG, mandibular | | • |  | € |  | Carina | • |  | € |
|  | |  |  |  |  |  |  |  |  |
|  | | | | | | | | | |
| a | Davidson’s fluid | | | | | | | | |
| b | Lungs were infused with 10% neutral buffered formalin | | | | | | | | |
| c | Lungs were weighed with trachea | | | | | | | | |
| d | Bouin’s fluid | | | | | | | | |
|  |  | | | | | | | | |
| LN | Lymph node | | | | | | | | |
| SG | Salivary gland | | | | | | | | |
| SC | Spinal cord | | | | | | | | |
| € | Examined microscopically | | | | | | | | |
|  |  | | | | | | | | |
| **Notes:** | | | | | | | | | |
| Paired organs weighed together. | | | | | | | | | |

**Histopathology Procedures**

Slide Preparation

As detailed in the Tissue Preservation section, tissues were prepared for microscopic examination by embedding in paraffin wax, sectioning, and staining with hematoxylin and eosin. Histological processing was conducted for all animals.

Histopathological Examination

Histopathological examination was performed on all animals as detailed in the Tissue Preservation section for Group 2 and 4 animals. Histopathological examination was also performed on esophagus, lungs, duodenum, ileum, jejunum, stomach, trachea, nasal cavity, nasopharynx, and carina for all Group 1 and 3 animals.

Mammary glands of all saline-control and high-dose animals and parathyroid of animals 2003A, 2501A, 2503A, 4002B, 4004C, 4005C, 4504C, and 4505C were not examined as they were too small.

**Toxicokinetic Analysis**

TK analysis was performed in WinNonlin Enterprise v5.2 [1]. TK parameters were calculated using NCA with NCA model 200 (Extravascular input). All tables and figures were completed using Microsoft Office (Excel) 2003 [2], SigmaPlot v9.0.1 [3], and WinNonlin AutoPilot [4] v1.1.1.

**Statistical Analyses**

Descriptive statistics (N, mean, SE, minimum, median, maximum, and %CV for concentrations and parameters, geometric mean and geometric %CV for TK parameters only) were calculated using WinNonlin AutoPilot or Excel and reported as appropriate (minimal sample size of 1 for mean, minimum, median, maximum, and 3 for variance derived statistics). Data were reported to three significant figures (or two decimal places for sampling time measurements and time observed parameters and individual concentrations) for values under 100. Values over 100 were reported to the nearest integer, when possible. Geometric %CV and %CV were reported to one decimal for all TK parameters. Due to software limitations, this could lead to the reporting of additional significant figures. Figures presenting mean concentration and mean TK parameter values were presented as mean ± SE.

An analysis of variance (ANOVA) was performed on the ln-transformed AUC_0-t_ and C_max_

parameters of glucagon calculated from the uncorrected concentration-time profiles. The

ANOVA model included gender as a fixed effect. A 10% level of significance was used to test the gender effect. The above statistical analyses were performed using WinNonlin v.5.2 [1] (LinMix module). Consistent with the 2 one-sided tests for bioequivalence, 90% confidence intervals (CI) for the ratio of geometric least squares means (LSMs) were calculated (male vs. female). A gender effect was concluded if the 90% CIs of the ratios of LSMs were contained within the 80%-125% range.

# 3. Single-Dose Intra-Tracheal Insufflation Toxicity in Rats

**Test System**

Species: Rat (*Rattus norvegicus*)

Strain: Sprague-Dawley Crl:CD (SD)

Source: Charles River Canada Inc., 188 rue Lasalle, St-Constant, Quebec, Canada

Total Animal No. in Study: 32 (16 males, 16 females) and 4 spares/sex

Body Weight Range: 290-363 g for males and 193-228 g for females at onset of treatment

Age Range: 8 weeks at onset of treatment

Acclimation Period: 2 weeks minimum

Allocation to Study Groups

During the acclimation period, 16 male and 16 female rats were assigned to their respective dose groups by block randomization based on body weights.

Animal Replacement

There was no animal replacement during the acclimation period. Four Group 2 animals were replaced following dosing due to low output of the intra-tracheal devices.

Administration of the Test and Control Articles

Prior to air or test-article insufflation, the animals’ food was removed for approximately 10 min. This procedure was taken to avoid the presence of food in the animals’ mouth/throat during the procedure.

Animals were administered either air or test article by pulmonary insufflation as a single dose. Prior to pulmonary insufflation of the air control or test article, each rat was anesthetized by inhalation of a mixture of 3.5% gaseous isoflurane in oxygen. The opening of the trachea was observed with an otoscope inserted in the animal’s mouth, and the insufflator was inserted in the trachea. Anesthetized animals were dosed by intra-tracheal administration using a PennCentury^®^ Intratracheal Dry Powder Insufflator (Model DP-4). The dose was approximately 0.5 mg/animal administered, and appropriate amount was loaded in the insufflator to ensure appropriate quantity was delivered to the animals. Three puffs of air were given per dose to expel as much test article as possible from the insufflator. Based on pre-treatment device delivery efficiency test performed, it was determined that approximately 1 mg GNP was required to deliver approximately 0.5 mg. The insufflator chambers were weighed prior to and after dosing to confirm actual dose delivered. The animals were closely observed during and following anesthetization associated with the intra-tracheal insufflation.

**In-life Observations**

Mortality

Mortality checks were performed at least once per day during all phases of the study.

Clinical Observations

For all main and recovery animals, cage-side clinical signs (ill health, behavioral changes, etc.) were recorded at least once daily during the acclimation and recovery periods and twice per day (pre- and post-administration) on the day of dosing.

Body Weights

Body weights were recorded for all animals at least once prior to group assignment (approximately 1 week prior to initiation of treatment).

The animals were weighed on Day -1, weekly during the recovery period, and prior to necropsy (fasted).

Food Consumption

Individual food intake was recorded for all main and recovery animals weekly starting the last week of the pre-treatment period.

**Necropsy Procedures**

Gross Examination and Organ Weights

Following an overnight fast, all main study animals were euthanized the day after dosing. All recovery animals were euthanized at completion of the 14-day recovery period following an overnight fast. The animals were anesthetized with sodium pentobarbital followed by exsanguination. To avoid autolytic changes, the necropsy examination of the carcass was conducted as soon as possible, on all animals that were euthanized at the study conclusion.

Gross pathology consisted of an external examination, including identification of all clinically recorded lesions, as well as a detailed internal examination. A staff pathologist was available for consultation during all necropsies performed during regular working hours.

For main and recovery animals, organs identified in the tissue preservation section were dissected, trimmed free of fat, and weighed. Organ weights relative to body weight were calculated.

Tissue Preservation

On completion of the gross pathology examination and selected organ weighing, the tissues and organs noted below were retained. Neutral buffered 10% formalin was used for fixation and preservation unless otherwise indicated.

| **ORGANS/TISSUES** | | **Retained**  **(**•**)** | **Weighed**  **( )** | **Examined**  **(**€**)** |  | **ORGANS/TISSUES** | **Retained**  **(**•**)** | **Weighed**  **( )** | **Examined**  **(**€**)** |
| --- | --- | --- | --- | --- | --- | --- | --- | --- | --- |
| Adrenals | | • | 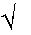 |  |  | Duodenum | • |  |  |
| Animal identification | | • |  |  |  | Jejunum | • |  |  |
| Aorta (thoracic) | | • |  |  |  | Ileum | • |  |  |
| Bone marrow smears (3) | | • |  |  |  | SC, cervical | • |  |  |
| Brain | | • | 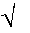 |  |  | Spleen | • | 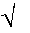 |  |
| Cecum | | • |  |  |  | Sternum and marrow | • |  |  |
| Colon | | • |  |  |  | Stomach | • |  |  |
| Epididymides | | •d |  |  |  | Testes | •d | 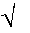 |  |
| Esophagus | | • |  |  |  | Thymus | • | 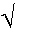 |  |
| Eyes | | •a |  |  |  | Thyroid gland/parathyroids | • | 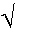 |  |
| Femur and marrow | | • |  |  |  | Tongue | • |  |  |
| Heart | | • | 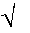 |  |  | Trachea | •c |  | € |
| Kidneys | | • | 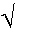 |  |  | Urinary bladder | • |  |  |
| Liver (2 lobes) | | • | 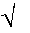 |  |  | Uterus | • | 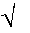 |  |
| Lungs (2 lobes) | | •bc | 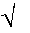 | € |  | Vagina | • |  |  |
| LN, mandibular | | • |  |  |  |  |  |  |  |
| LN, mesenteric | | • |  |  |  | Abnormal findings | • |  | € |
| Mammary gland (inguinal) | | • |  |  |  |  |  |  |  |
| Optic nerves | | •a |  |  |  |  |  |  |  |
| Ovaries | | • | 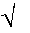 |  |  | **Additional tissues presented below** | | | |
| Pancreas | | • |  |  |  |  |  |  |  |
| Pituitary | | • | 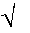 |  |  | Nasopharynx | • |  | € |
| Prostate | | • | 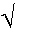 |  |  | Nasal cavity | • |  | € |
| Rectum | | • |  |  |  | Larynx | • |  | € |
| SG, mandibular | | • |  |  |  | LN, tracheo-bronchial | • |  | € |
| Sciatic nerve | | • |  |  |  | Carina | • |  | € |
| Seminal vesicles | | • |  |  |  |  |  |  |  |
| Skeletal muscle | | • |  |  |  |  |  |  |  |
| Skin and subcutis (inguinal) | | • |  |  |  |  |  |  |  |
|  | | | | | | | | | |
| a | Davidson’s fluid | | | | | | | | |
| b | Lungs were infused with 10% neutral buffered formalin | | | | | | | | |
| c | Lungs were weighed with trachea and recorded in Provantis as lungs in organ weight record | | | | | | | | |
| d | Bouin’s fluid | | | | | | | | |
|  |  | | | | | | | | |
|  |  | | | | | | | | |
| LN | Lymph node | | | | | | | | |
| SG | Salivary gland | | | | | | | | |
| SC | Spinal cord | | | | | | | | |
| € | Examine microscopically | | | | | | | | |
|  |  | | | | | | | | |
| **Notes:** | | | | | | | | | |
| Paired organs weighed together. | | | | | | | | | |

**Histopathology Procedures**

Slide Preparation

As detailed in the Tissue Preservation section, tissues were prepared for microscopic examination by embedding in paraffin wax, sectioning, and staining with hematoxylin and eosin. Histological processing was conducted for all tissues detailed in the Tissue Preservation section for animals detailed below.

Histopathological Examination

Histopathological examination was performed on the respiratory tract tissues (nasal cavities, nasopharynx, larynx, trachea, tracheo-bronchial lymph node, lungs, and carina) as well as abnormal findings of all animals.

# 4. Single-Dose Ocular Tolerance in Rabbits

**Test System**

Four male New Zealand white rabbits (*Oryctolagus cuniculus*), including 1 spare animal, were received from Charles River Canada Inc. (324 St-Régis Nord, C.P. 400, St-Constant, Quebec, Canada). At the onset of dosing, the age of the animals was between 3 months and 10 days, to 3 months and 13 days, and the body weights ranged from 3.0 to 3.1 kg.

Animal Assignment/Replacement

For logistical reasons, the animals were assigned to replicate sub-groups, for which dosing was given on different days. Following the start of dosing, animal 1002 was replaced (by a spare animal from the same shipment, maintained under the same environmental conditions) due to receiving approximately only 43% (12.8 mg) of the targeted 30-mg dose following post-dose calculations. Only the data of the replacement animal (1102) were reported. The spare animal was released from the study following the replacement period.

Preparation of the Test System

Within 24 h before treatment, both eyes of each animal were examined with a slit lamp and an inspection lamp fitted with white and UV-light and magnifying glass to ensure that there were no defects or irritation prior to testing. The examination was performed before and after instillation of fluorescein solution (fluorescein sodium and benoxinate HCl, 0.25/0.4% solution). Approximately two drops of fluorescein solution were instilled in both eyes. On the day of dosing administration, the animals were provided with a subcutaneous injection of an analgesic (buprenorphine, 0.2 mL/animal), between 50 min and 1 h and 22 min prior to dosing. Dose levels of the analgesic employed in the preparation of the animals were in accordance with the facility’s standard operating procedures.

**In-life Observations**

Mortality

Mortality checks were recorded concomitantly with the cage-side clinical observations (see below), during all phases of the study.

Clinical Observations

Cage-side clinical signs (ill health, behavioral changes, etc.) were recorded twice daily starting 1 day following animal arrival and during the dosing and observation periods. Additional clinical observations were performed as necessary. DCE was performed on each animal on the day of animal assignment, during the week prior to initiation of treatment, 1 day prior to initiation of treatment, and on the day of necropsy.

Ocular Observations

Both treated (left) and untreated (right) eyes were examined by the clinical veterinarian, with a slit lamp and an inspection lamp fitted with white and UV-light and a magnifying glass. An evaluation of ocular irritation was performed on all animals within 24 h before treatment (see Preparation of the Test System section), prior to dosing, and at 1, 24, 48, and 72 h post-dose. To evaluate the possibility of a delayed effect, ocular lesions were scored both before and after the fluorescein solution instillation at each time point. Approximately two drops of fluorescein solution were instilled in both eyes at each time point, with the exception of animal 1003 (number of fluorescein drops not recorded) at the 48-h post-time point observation. The objective of the procedure was attained without confirmation of the number of fluorescein drops for this animal, therefore, it has no impact on the study. The ocular observations were terminated following the 72-h post-dose, since there was no persistent corneal lesion or evidence of irritation that required further examination.

The following subjective numerical scoring system was used to grade ocular reactions to the treatment:

Ocular Grading

| **Opacity: degree of density**  **(Area most dense taken for reading)** | **Score** |
| --- | --- |
| No ulceration or opacity | 0 |
| Scattered or diffuse area of opacity (other than slight dulling of normal luster),  details of iris clearly visible | 1 |
| Easily discernible translucent area, details of iris slightly obscured | 2 |
| Nacrous areas, no details of iris visible, size of pupil barely discernible | 3 |
| Opaque cornea, iris not discernible through the opacity | 4 |

Cornea

| **Area of cornea involved** | **Score** |
| --- | --- |
| Cornea not involved | 0 |
| One quarter (or less), but not zero | 1 |
| More than 1 quarter, but less than half | 2 |
| More than half, but less than 3 quarters | 3 |
| More than 3 quarters, up to whole area | 4 |

Iris

|  | **Score** |
| --- | --- |
| Normal | 0 |
| Markedly deepened rugae, congestion, swelling, moderate circumcorneal hyperemia, or injection, iris reactive to light (a sluggish reaction is considered to be an effect) | 1 |
| Hemorrhage, gross destruction, or no reaction to light | 2 |

Conjunctivae

| **Redness (refers to palpebral and bulbar conjunctivae, excluding cornea and iris)** | **Score** |
| --- | --- |
| Normal | 0 |
| Some blood vessels hyperemic (injected) | 1 |
| Diffuse, crimson color, individual vessels not easily discernible | 2 |
| Diffuse beefy red | 3 |

Chemosis

| **Swelling (refers to lids and/or nictating membranes)** | **Score** |
| --- | --- |
| Normal | 0 |
| Some swelling above normal | 1 |
| Obvious swelling, with partial eversion of lids | 2 |
| Swelling, with lids about half closed | 3 |
| Swelling, with lids more than half closed | 4 |

Body Weights

Body weights were recorded for all animals on the day of animal assignment, during the week prior to initiation of treatment, 1 day prior to initiation of treatment, and on the day prior to necropsy.

**Terminal Procedures**

Macroscopic Examination

Animals surviving to scheduled termination were euthanized following an overnight period without food and subjected to a macroscopic examination on Day 4. Animals were euthanized by an intravenous overdose of sodium pentobarbital, followed by exsanguination. For all animals, necropsy consisted of an external macroscopic examination limited to the eyes and eyelids, which were discarded following the examination. To avoid autolytic changes, the macroscopic examination of the carcass was conducted as soon as possible on all animals at scheduled termination.

Histopathology

No histopathological preparation or evaluation was performed.

**References**

1. WinNonlin® Enterprise Edition software v5.2. Mountain View, CA: Pharsight.
2. Microsoft® Office (Excel) 2003. Redmond, WA: Microsoft Corporation.
3. SigmaPlot® v9.0.1. San Jose, CA: Systat Corporation.
4. WinNonlin® AutoPilot™ v1.1.1. Mountain View, CA: Pharsight.
5. Nedelman JR, Jia X. An extension of Satterthwaite's approximation applied to pharmacokinetics. J Biopharm Stat. 1998;8:317-25.
6. Holder DJ. Comments on Nedelman and Jia's extension of Satterthwaite's approximation applied to pharmacokinetics. J Biopharm Stat. 2001;11:75-9.
